# Modelling COVID-19 pandemic control strategies in metropolitan and rural health districts in New South Wales, Australia

**DOI:** 10.1038/s41598-023-37240-8

**Published:** 2023-06-26

**Authors:** Azizur Rahman, Md Abdul Kuddus, Ryan H. L. Ip, Michael Bewong

**Affiliations:** 1 School of Computing, Mathematics and Engineering, Charles Sturt University, Wagga Wagga, NSW 2678 Australia; 2 Australian Institute of Tropical Health and Medicine, James Cook University, Townsville, QLD 4811 Australia; 3 Department of Mathematics, University of Rajshahi, Rajshahi, 6205 Bangladesh

**Keywords:** Infectious diseases, Public health, Health policy

## Abstract

COVID-19 remains a significant public health problem in New South Wales, Australia. Although the NSW government is employing various control policies, more specific and compelling interventions are needed to control the spread of COVID-19. This paper presents a modified SEIR-X model based on a nonlinear ordinary differential equations system that considers the transmission routes from asymptomatic (Exposed) and symptomatic (Mild and Critical) individuals. The model is fitted to the corresponding cumulative number of cases in metropolitan and rural health districts of NSW reported by the Health Department and parameterised using the least-squares method. The basic reproduction number $({\mathrm{R}}_{0})$, which measures the possible spread of COVID-19 in a population, is computed using the next generation operator method. Sensitivity analysis of the model parameters reveals that the transmission rate had an enormous influence on ${\mathrm{R}}_{0}$, which may be an option for controlling this disease. Two time-dependent control strategies, namely preventive (it refers to effort at inhibiting the virus transmission and prevention of case development from Exposed, Mild, Critical, Non-hospitalised and Hospitalised population) and management (it refers to enhance the management of Non-hospitalised and Hospitalised individuals who are infected by COVID-19) measures, are considered to mitigate this disease’s dynamics using Pontryagin’s maximum principle. The most sensible control strategy is determined through the cost-effectiveness analysis for the metropolitan and rural health districts of NSW. Our findings suggest that of the single intervention strategies, enhanced preventive strategy is more cost-effective than management control strategy, as it promptly reduces COVID-19 cases in NSW. In addition, combining preventive and management interventions simultaneously is found to be the most cost-effective. Alternative policies can be implemented to control COVID-19 depending on the policymakers’ decisions. Numerical simulations of the overall system are performed to demonstrate the theoretical outcomes.

## Introduction

COVID-19, the disease caused by the novel SARS-COV-2 virus, is still posing a heavy burden on the health systems in many countries all over the world. In Australia, New South Wales (NSW) and other states have been highlighted as settings at particular risk of COVID-19 spread^1^. Not only are they an ‘enclosed society’ characterized by a high degree of contact among people, but persons deprived of their liberty have little means to implement the necessary measures of COVID-19 prevention such as social distancing, hand hygiene and isolation.

**Figure 1:**
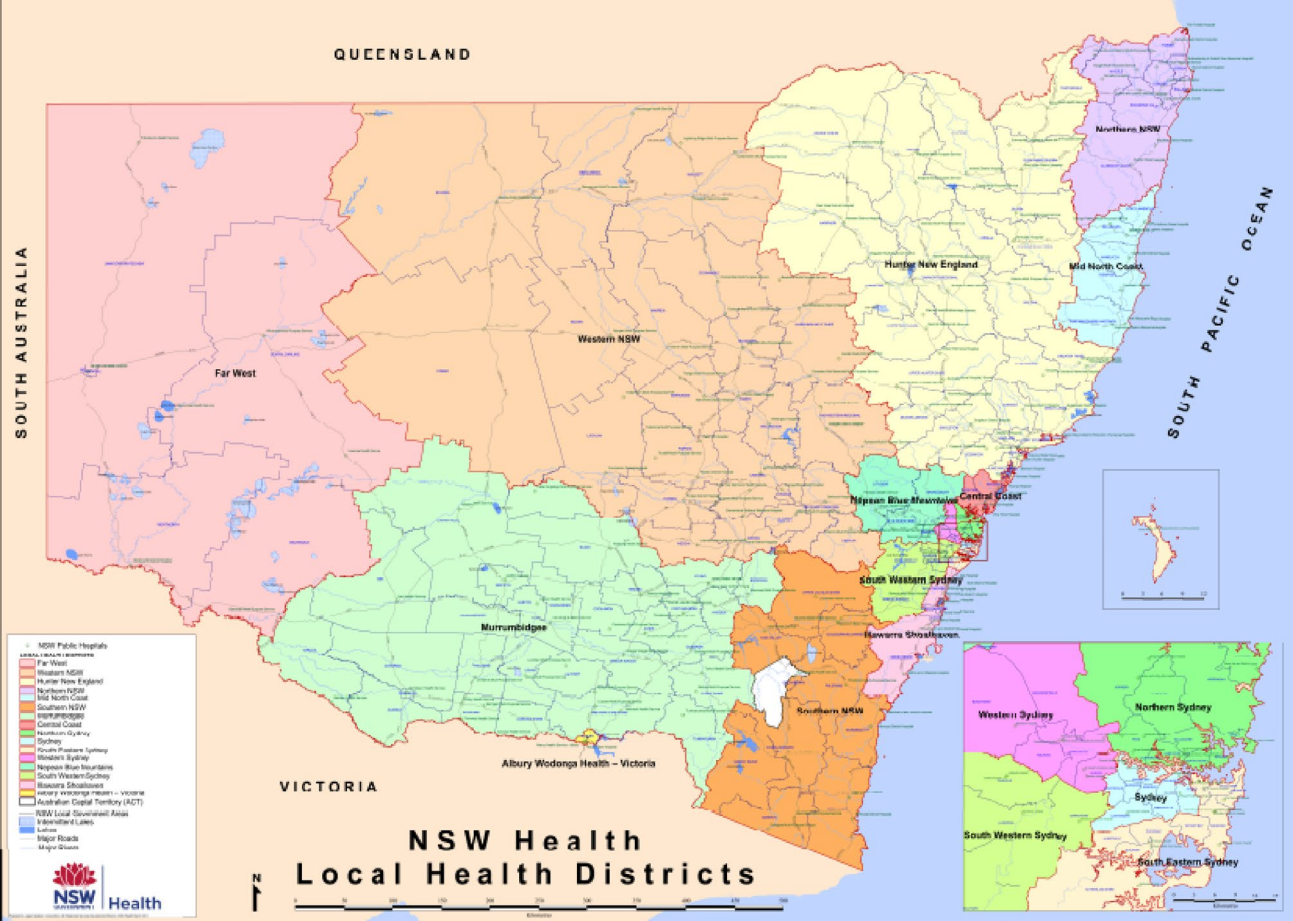
The map displays the locations and distributions of metropolitan and rural health districts in NSW Australia. (Source: https://www.health.nsw.gov.au/lhd/Documents/lhd-wall-map.pdf).

**Figure 2:**
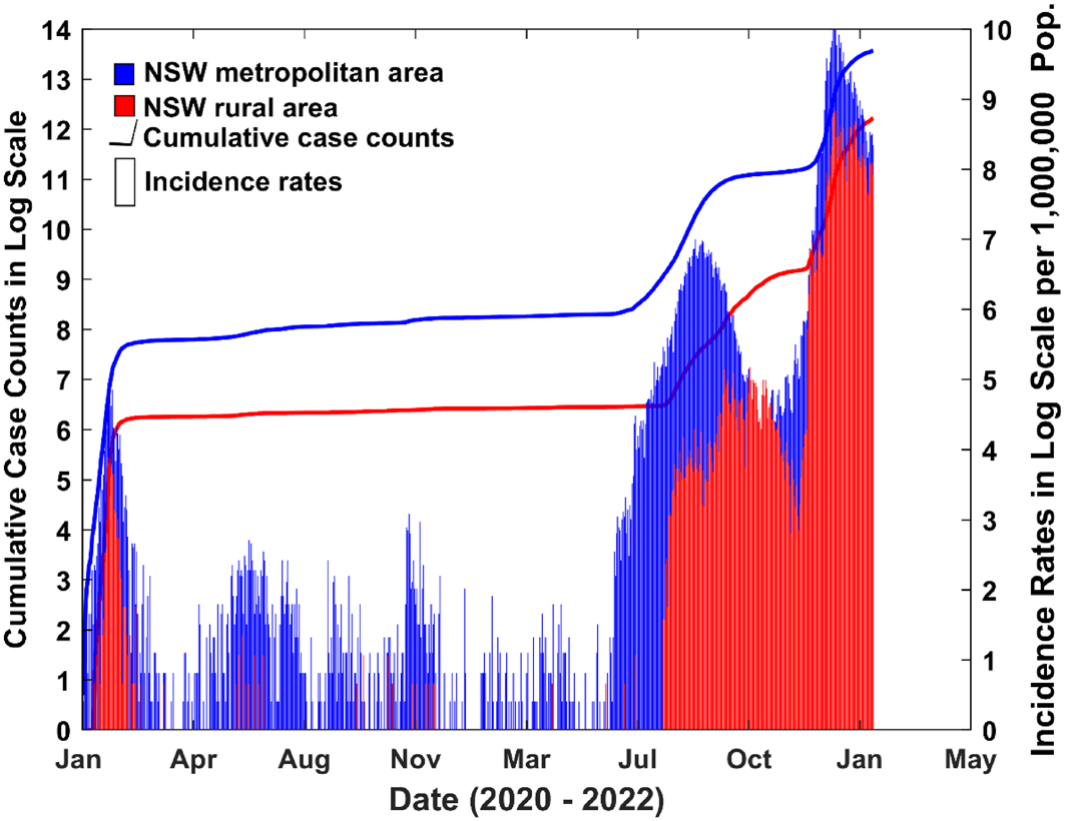
COVID-19 case counts and incidence rates (per one million population) in NSW metropolitan and rural areas (red bars indicate rural cases and blue bars indicate metropolitan cases) (Data source: https://www.health.nsw.gov.au/lhd/Pages/default.aspx).

There are six states in Australia, NSW is one of them and it is situated on the east coast of Australia. NSW connects Queensland to the north, Victoria to the south, South Australia to the west and encloses the entire of the Australian Capital Territory (see Fig. 1). NSW is formally divided into two local health districts: metropolitan and rural. The metropolitan area includes eight local health districts such as Central Coast, Illawarra Shoalhaven, Nepean Blue Mountains, Northern Sydney, South Eastern Sydney, South Western Sydney, Sydney and Western Sydney^2^. Seven local health districts such as Far West, Hunter New England, Mid North Coast, Murrumbidgee, Northern NSW, Southern NSW and Western NSW cover the rural NSW region^2^. Fig. 2 shows the trends of cumulative cases in metropolitan and rural districts in NSW from January 2020 to February 2022. It can be observed that the cumulative cases and incidences rates are higher in metropolitan health districts compared to rural districts.

Concerns about COVID-19 occurring in places of positive test results have been made real from numerous outbreaks that have already taken place throughout the pandemic^3^. Many countries have responded to the threat by diverting persons away from COVID-19 test centres. However, peer-reviewed literature regarding COVID-19 and places of positive test results is restricted to opinion pieces or commentaries. These universally emphasize the vulnerabilities of COVID-19 test centres during this pandemic. However, they make no attempt to enumerate the potential cause and consequences of an outbreak, nor set targets for inmate reductions. COVID-19 outbreaks in COVID-19 test centres (e.g. in China, Canada, and the US) have largely gone unremarked in the peer-reviewed literature. In contrast, the ‘super spreader’ event onboard the Diamond Princess cruise ship has been the subject of numerous epidemiological publications^4^ leading to many lessons learnt.

Experience with respiratory outbreaks and other diseases have repeatedly demonstrated that no matter how isolated a community is, it is not necessarily insulated from infection. Even where there are no complicating factors, such as the age distribution or the presence of individuals with greater susceptibility in the enclosed population, their organization tends to increase transmission and secondary infection risk^5^. In this way, conditions of positive test results have been described as ‘amplifiers of infectious disease’^6^. Numerous outbreaks, particularly reports of influenza^7^ and tuberculosis^8^ demonstrate the propensity for outbreaks to spread rapidly.

Modelling has been utilised as a tool to address gaps in knowledge and inform detention health policies in the prevention and control of infectious disease in detention settings^9^. Researchers have been using various models to provide insights and guide interventions to control the spread of COVID-19. These have been undertaken in various settings such as the epicentre of Wuhan, China^10^, in high-income countries to predict health service needs^11,12^, in middle-income countries to check the effects of total population and population densities^13,14^ to refugee camps and other low-income settings^15,16^.

On the 10^th^ of February 2022, 988,357 out of the 2,433,278 COVID-19 cumulative cases (around 40%) in Australia occurred in NSW^17^. Strong contingency planning is needed so that health authorities can act accordingly^18^. To this end, mathematical modelling offers an alternative source of information to enlighten preparedness and preventative interventions. Given the differences between metropolitan and rural areas in NSW, a model that allows different parameters under different control settings and locations is needed. Motivated by the continuing and distinctive spread of COVID-19 infections in metropolitan and rural areas of NSW (see Fig. 2), this study is aimed at developing a model that can be used to represent the outbreak of COVID-19 in NSW, hence providing practical recommendations for health and justice authorities to support preparedness measures to better protect the vulnerable population and surrounding communities.

We develop a novel nonlinear deterministic SEIR-X model, or the SEMCNHRD model, for COVID-19 incorporating transmission routes from two infectious classes, including mild and critical individuals, which is parameterized and analyzed based on the cumulative number of reported cases. The model behaviour over a long period is examined qualitatively and quantitatively. Sensitivity analysis is used to assess the impact of fluctuations in model parameters for the epidemiological threshold on the formulation of strategies required to control the spread of the disease. Further, the model can be used to derive the optimal control strategy and the optimal levels of time-dependent preventive and management procedures to be implemented in metropolitan and rural districts to diminish the number of cases in the population effectively. The cost-effective intervention, which combines both preventive and management control strategies, capable of flattening the number of cases curve over a fixed time interval, is recommended and compared between metropolitan and rural health districts of NSW.

The remainder of this paper is structured as follows. In “Methods and materials” section, we present the SEIR-X model with differential infectivity, the model fitting procedures and the sensitivity analysis of the model outputs. “Optimal control strategy and analysis” section reports the optimal control strategy and analysis results. Finally, in “Discussion”, we provide a summary of significant findings, discuss their importance for public health policy making and propose guidelines for future efforts.

## Methods and materials

### Model development

The following model, which we call the SEIR-X model, is proposed for modelling the transmission of COVID-19. The model consists of the following mutually exclusive compartments: Susceptible $\mathrm{S}(\mathrm{t})$, uninfected individuals who are susceptible to the COVID-19 infection; Exposed $\mathrm{E}(\mathrm{t})$, those who are infected but have not yet entered the active COVID-19 stage; $\mathrm{M}\left(\mathrm{t}\right)$, Mild individuals who are infected, infectious and have mild respiratory illness symptoms such as nasal congestion, runny nose and a sore throat; $\mathrm{C}\left(\mathrm{t}\right)$, Critical individuals who are infected, infectious and have severe symptoms including shortness of breath, chest discomfort and bluish face. After seeking medical advice, critical individuals are classified as either ${\mathrm{N}}_{\mathrm{H}}$—non-hospitalised individuals but still infected, and $\mathrm{H}(\mathrm{t})$—hospitalised individuals who are still infected. The last two compartments are the Recovered $\mathrm{R}(\mathrm{t})$, who were previously infected and were successfully treated, and Death $\mathrm{D}(\mathrm{t})$. At the same time t, an individual is classified into one and only one compartment. The total population size $\mathrm{N}(\mathrm{t})$ is assumed to be a constant at time t and well mixed:

1 $$\mathrm{N}\left(\mathrm{t}\right)=\mathrm{S}\left(\mathrm{t}\right)+\mathrm{E}\left(\mathrm{t}\right)+\mathrm{M}\left(\mathrm{t}\right)+\mathrm{C}\left(\mathrm{t}\right)+{\mathrm{N}}_{\mathrm{H}}\left(\mathrm{t}\right)+\mathrm{H}\left(\mathrm{t}\right)+\mathrm{R}\left(\mathrm{t}\right)+\mathrm{D}\left(\mathrm{t}\right).$$

**Figure 3:**
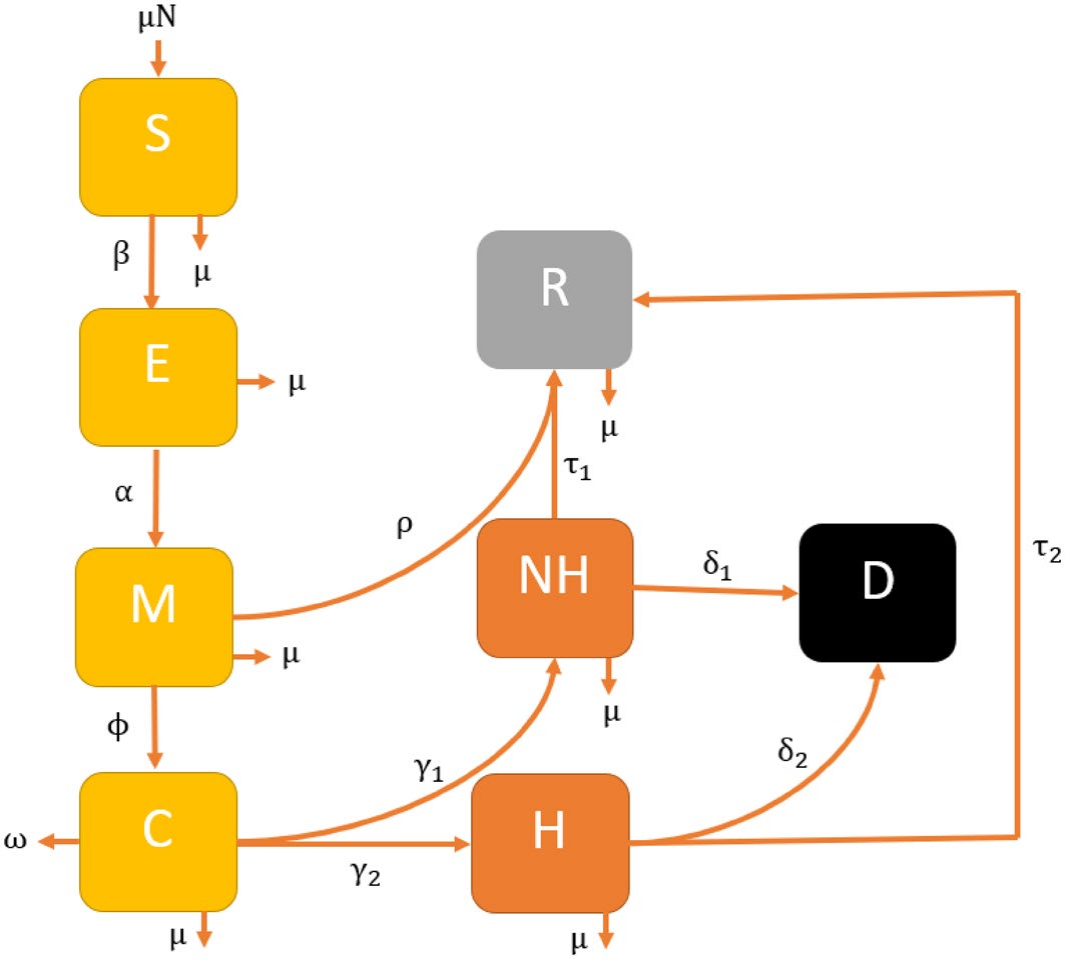
The SEIR-X (SEMCNHRD) model structure: the population is divided into the following eight classes: susceptible, exposed (and not yet symptomatic), infectious (symptomatic) i.e. mild (mild or moderate symptom) and critical (severe symptom), death and recovered (i.e. isolation, recovered, or otherwise non-infectious).

To ensure the population size remains constant, we replace all deaths by newborns in the susceptible class. This includes deaths through natural causes, which occur in all states at a constant rate $\upmu$, and COVID-19 related deaths which occur at a constant rate $\upomega$. Susceptible individuals may be infected with a circulating strain of COVID-19 at the rate $\lambda =\upbeta \left(\mathrm{M}\left(\mathrm{t}\right)+\mathrm{C}(\mathrm{t})\right)$ and move to the corresponding exposed class $\mathrm{E}(\mathrm{t})$. Here, $\upbeta$ is the probability of susceptible individual contracts infection after contact with Mild or Critical individuals with COVID-19. Those with latent infection progress to mild (the M(t) class) due to reactivation of the latent infection at an average period $\mathrm{\alpha }$. However, some Mild individuals move to the recovery class $\mathrm{R }(\mathrm{t})$ at an average period $\uprho$ due to natural recoveries and the rest of the Mild class individuals move to the critical compartment at an average period $\upphi$ due to the progression and possibly comorbidities with other diseases, including hypertension, diabetes, cardiovascular disease, and respiratory system disease^19^. A proportion (type of ratio) of the Critical individuals move to the Non-hospitalised and Hospital compartments at an average period ${\upgamma }_{1}$ and ${\upgamma }_{2}$, respectively. Some Non-hospitalised individuals progress to the recovered compartment $\mathrm{R}\left(\mathrm{t}\right)$ at an average period ${\uptau }_{1}$ through treatment and the rest progress to the death compartment (D) at an average period ${\updelta }_{1}$. Similarly, some of the Hospitalised individuals progress to the recovered compartment $\mathrm{R}\left(\mathrm{t}\right)$ at an average period ${\uptau }_{2}$ through treatment and the rest progress to the death compartment (D) at an average period ${\updelta }_{2}$. A flow diagram of our proposed model is presented in Fig. 3.

In this case, the model can be expressed by the following deterministic system of nonlinear ordinary differential equations:

2 $$\frac{\mathrm{dS}}{\mathrm{dt}}=\mathrm{\mu N}-\upbeta \left(\mathrm{M}+\mathrm{C}\right)\mathrm{S}-\mathrm{\mu S}$$

3 $$\frac{\mathrm{dE}}{\mathrm{dt}}=\upbeta \left(\mathrm{M}+\mathrm{C}\right)\mathrm{S}-(\mathrm{\alpha }+\upmu )\mathrm{E}$$

4 $$\frac{\mathrm{dM}}{\mathrm{dt}}=\mathrm{\alpha E}-(\upphi +\upmu +\uprho )\mathrm{M}$$

5 $$\frac{\mathrm{dC}}{\mathrm{dt}}=\mathrm{\phi M}-(\upomega +\upmu +{\upgamma }_{1}+{\upgamma }_{2})\mathrm{C}$$

6 $$\frac{{\mathrm{dN}}_{\mathrm{H}}}{\mathrm{dt}}={\upgamma }_{1}\mathrm{C}-\left(\upmu +{\updelta }_{1}+{\uptau }_{1}\right){\mathrm{N}}_{\mathrm{H}}$$

7 $$\frac{\mathrm{dH}}{\mathrm{dt}}={\upgamma }_{2}\mathrm{C}-(\upmu +{\updelta }_{2}+{\uptau }_{2})\mathrm{H}$$

8 $$\frac{\mathrm{dR}}{\mathrm{dt}}=\mathrm{\rho M}+{\uptau }_{1}{\mathrm{N}}_{\mathrm{H}}+{\uptau }_{2}\mathrm{H}-\mathrm{\mu R}$$

9 $$\frac{\mathrm{dD}}{\mathrm{dt}}={\updelta }_{1}{\mathrm{N}}_{\mathrm{H}}+{\updelta }_{2}\mathrm{H}$$

Given the non-negative initial conditions for the system above, it is straightforward to show that each of the state variables remains non-negative for all $\mathrm{t}>0.$ Moreover, summing Eqs. (2)–(9) we find that the size of the total population, $\mathrm{N}(\mathrm{t})$ satisfies i.e. $\mathrm{N}\left(\mathrm{t}\right)=\mathrm{constant}$.

This shows that the total population size $\mathrm{N}(\mathrm{t})$ is a constant and it naturally follows that each of the compartment states $\mathrm{S},\mathrm{E},\mathrm{ M},\mathrm{ C}, {\mathrm{N}}_{\mathrm{H}},\mathrm{ H},\mathrm{ R and D}$ is bounded. Given the positivity and boundedness of the system solutions, the feasible region for Eqs. (2)–(9) is given by

$${\mathrm{D}}_{1}=\left\{ \left(\mathrm{S},\mathrm{ E},\mathrm{ M},\mathrm{ C}, {\mathrm{N}}_{\mathrm{H}},\mathrm{ H},\mathrm{ R},\mathrm{ D}\right)\in {\mathbb{R}}_{+}^{8} :\mathrm{S}+\mathrm{E}+\mathrm{M}+\mathrm{C}+{\mathrm{N}}_{\mathrm{H}}+\mathrm{H}+\mathrm{R}+\mathrm{D}=\mathrm{N}\right\},$$

where ${\mathrm{D}}_{1}$ is positively invariant.

### Basic reproduction number

The basic reproduction number (${\mathrm{R}}_{0}$) is defined as the expected number of secondary cases created by a single infectious case introduced into a totally susceptible population. The disease can spread in a population only if the basic reproduction number is greater than one. An epidemic occurs when an infection spreads through and infects a significant proportion of a population. A disease-free population is possible when the basic reproduction number is less than one, which means that the disease naturally fades-out^20,21^.

There are eight states in the modelling system in which five belong to the infected states, i.e. $\mathrm{E},\mathrm{ M},\mathrm{ C}, {\mathrm{N}}_{\mathrm{H}}$ and $\mathrm{H}$, and three are uninfected states, i.e. $\mathrm{S},\mathrm{ R}$ and $\mathrm{D}$. At the infection-free steady-state $\mathrm{E}=\mathrm{M}=\mathrm{C}={\mathrm{N}}_{\mathrm{H}}=\mathrm{H}=\mathrm{R}=\mathrm{D}=0,$ hence ${\mathrm{S}}_{0}=\mathrm{N},$ where ${S}_{0}$ is the initial susceptible population. The Eqs. (3)–(7) are closed, in that they do not involve the derivation of $\mathrm{S}$ from steady state value. Also, $\mathrm{R}$ and $\mathrm{D}$ do not appear in Eqs. (3)–(7)$,$ and for $(\mathrm{E},\mathrm{M},\mathrm{C},{\mathrm{N}}_{\mathrm{H}},\mathrm{H})$ we have the following equations:

10 $$\frac{\mathrm{dE}}{\mathrm{dt}}=\upbeta \left(\mathrm{M}+\mathrm{C}\right)\mathrm{S}-(\mathrm{\alpha }+\upmu )\mathrm{E}$$

11 $$\frac{\mathrm{dM}}{\mathrm{dt}}=\mathrm{\alpha E}-(\upphi +\upmu +\uprho )\mathrm{M}$$

12 $$\frac{\mathrm{dC}}{\mathrm{dt}}=\mathrm{\phi M}-(\upomega +\upmu +{\upgamma }_{1}+{\upgamma }_{2})\mathrm{C}$$

13 $$\frac{{\mathrm{dN}}_{\mathrm{H}}}{\mathrm{dt}}={\upgamma }_{1}\mathrm{C}-\left(\upmu +{\updelta }_{1}+{\uptau }_{1}\right){\mathrm{N}}_{\mathrm{H}}$$

14 $$\frac{\mathrm{dH}}{\mathrm{dt}}={\upgamma }_{2}\mathrm{C}-(\upmu +{\updelta }_{2}+{\uptau }_{2})\mathrm{H}$$

Here, these Ordinary Differential Equations (ODEs) in (10)–(14) are referred to as the infection subsystem, as they only describe the production of newly infected individuals and changes in the states of already infected individuals.

By setting $\mathbf{x}={(\mathrm{E},\mathrm{M},\mathrm{C},{\mathrm{N}}_{\mathrm{H}},\mathrm{H})}^{\mathrm{^{\prime}}}$, where the prime denotes transpose, the infection subsystem can be written in the following form:

15 $$\dot{\mathbf{x}}=\left(\mathrm{T}+\Sigma \right)\mathbf{x}.$$

The matrix $\mathrm{T}$ corresponds to the transmission, and the matrix $\Sigma$ to transitions. All epidemiological events that lead to new infections are incorporated in the model via $\mathrm{T}$ and other events via $\Sigma$. If the infected states are indicated with $\mathrm{i}$ and $\mathrm{j}$ with $\mathrm{i},\mathrm{j}\in \{1, 2, 3, 4, 5\}$, then the entry ${\mathrm{T}}_{\mathrm{ij}}$ is the rate at which individuals in infected state $\mathrm{j}$ give rise to individuals in infected state $\mathrm{i}$. The matrices $\mathrm{T}$ and $\Sigma$ admit the form

$$\mathrm{T}=\left(\begin{array}{ccc}0&\upbeta {\mathrm{S}}_{0}& \begin{array}{ccc}\upbeta {\mathrm{S}}_{0}& 0& 0\end{array}\\ 0& 0& \begin{array}{ccc}0& 0& 0\end{array}\\ \begin{array}{c}0\\ 0\\ 0\end{array}& \begin{array}{c}0\\ 0\\ 0\end{array}& \begin{array}{c}\begin{array}{ccc}0& 0& 0\end{array}\\ \begin{array}{ccc}0& 0& 0\end{array}\\ \begin{array}{ccc}0& 0& 0\end{array}\end{array}\end{array}\right)\mathrm{and}$$

$$\Sigma = \left( {\begin{array}{*{20}l} { - \left( {{\upalpha } + {\upmu }} \right)} & 0 & 0 & 0 & 0 \\ \alpha & { - \left( {{\upphi } + {\uprho } + {\upmu }} \right)} & 0 & 0 & 0 \\ 0 & {\upphi } & { - \left( {{\upomega } + {\upgamma }_{1} + {\upgamma }_{2} + {\upmu }} \right)} & 0 & 0 \\ 0 & 0 & {{\upgamma }_{1} } & { - \left( {{\updelta }_{1} + {\uptau }_{1} + {\upmu }} \right)} & 0 \\ 0 & 0 & {{\upgamma }_{2} } & 0 & { - \left( {{\updelta }_{2} + {\uptau }_{2} + {\upmu }} \right)} \\ \end{array} } \right)$$

The next-generation matrix, $\mathrm{K}$, is given by^22^ (note the essential minus sign)

$${\text{K}} = - {\text{T}}\Sigma ^{{ - 1}} = {\text{T}}\left( { - \Sigma ^{{ - 1}} } \right) = \left( {\begin{array}{*{20}c} {{\text{A}}_{1} } & {{\text{A}}_{2} } & {{\text{A}}_{3} } & 0 & 0 \\ 0 & 0 & 0 & 0 & 0 \\ 0 & 0 & 0 & 0 & 0 \\ 0 & 0 & 0 & 0 & 0 \\ 0 & 0 & 0 & 0 & 0 \\ \end{array} } \right)$$

where

$${\mathrm{A}}_{1}=\frac{{\mathrm{S}}_{0}\mathrm{\alpha \beta }}{(\mathrm{\alpha }+\upmu )(\upphi +\uprho +\upmu )}+\frac{{\mathrm{S}}_{0}\mathrm{\alpha \beta \phi }}{(\mathrm{\alpha }+\upmu )(\upphi +\uprho +\upmu )({\upgamma }_{1}+{\upgamma }_{2}+\upomega +\upmu )}$$

$${\mathrm{A}}_{2}=\frac{{\mathrm{S}}_{0}\upbeta }{(\mathrm{\alpha }+\upmu )(\upphi +\uprho +\upmu )}+\frac{{\mathrm{S}}_{0}\mathrm{\beta \phi }}{(\mathrm{\alpha }+\upmu )(\upphi +\uprho +\upmu )({\upgamma }_{1}+{\upgamma }_{2}+\upomega +\upmu )}$$

$${\mathrm{A}}_{3}=\frac{{\mathrm{S}}_{0}\upbeta }{({\upgamma }_{1}+{\upgamma }_{2}+\upomega +\upmu )}$$

The dominant eigenvalue of $\mathrm{K}$ is the basic reproduction number for COVID-19, which represents the average number of new infections produced by one infected individual. Here, the basic reproduction number can be expressed as:${\mathrm{R}}_{0}=\frac{{\mathrm{S}}_{0}\mathrm{\alpha \beta }}{(\mathrm{\alpha }+\upmu )(\upphi +\uprho +\upmu )}+\frac{{\mathrm{S}}_{0}\mathrm{\alpha \beta \phi }}{(\mathrm{\alpha }+\upmu )(\upphi +\uprho +\upmu )({\upgamma }_{1}+{\upgamma }_{2}+\upomega +\upmu )}.$

The first term represents the probability of becoming infectious once infected with mean infectious period $\frac{1}{(\upphi +\uprho +\upmu )}$. The second term indicates the expected number of infected individuals due to the comorbidities with other diseases with mean infectious period $\frac{1}{(\upphi +\uprho +\upmu )({\upgamma }_{1}+{\upgamma }_{2}+\upomega +\upmu )}$.

We provide detailed analysis, including the existence and stability of the equilibrium points, for the proposed COVID-19 model (2)–(9) in the supplementary materials, see the sections existence of equilibria section and global stability of disease-free equilibrium.

### Parameter estimation and model fitting

**Figure 4:**
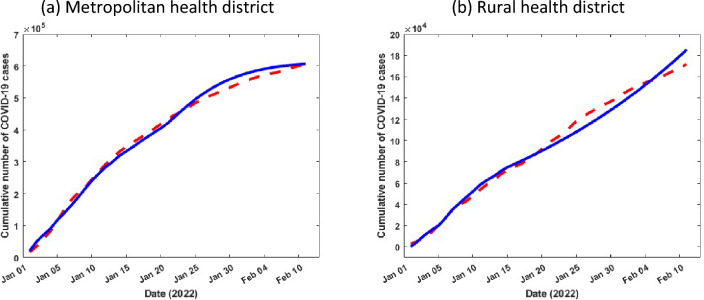
Cumulative confirmed COVID-19 cases data from January 01, 2022 to February 10, 2022 (red dash) and the corresponding model best fit (blue solid curve) in NSW.

We estimated the COVID-19 model parameters from fitting different combinations of parameters in Eqs. (2)–(9) to the actual reported cases in metropolitan and rural health districts in NSW^23^. In order to parameterize the model, we obtained some of the initial parameter values from literature (see Table 1), and others were estimated from data fitting. The estimation of parameters was carried out using the least squares method which minimises summation of the square errors given by $\sum {\left(\mathrm{Y}\left(\mathrm{t},\mathrm{q}\right)-{\mathrm{X}}_{\mathrm{real}}\right)}^{2}$ subject to the COVID-19 model (2)–(9), where ${\mathrm{X}}_{\mathrm{real}}$ is the real reported data, and $\mathrm{Y}(\mathrm{t},\mathrm{q})$ denotes the solution of the model corresponding to the number of cases over time $\mathrm{t}$ with the set of estimated parameters, denoted by $\mathrm{q}$. We assume the initial condition for the state variables in the following way: in metropolitan health district, $\mathrm{N}\left(0\right)=\mathrm{226,5170},\mathrm{ E}\left(0\right)=\mathrm{7,286},\mathrm{ M}\left(0\right)=910,\mathrm{ C}\left(0\right)=300, {\mathrm{N}}_{\mathrm{H}}\left(0\right)=100,\mathrm{ H}\left(0\right)=40,\mathrm{ R}\left(0\right)=10,\mathrm{ D}\left(0\right)=0,\mathrm{ S}\left(0\right)=\mathrm{N}\left(0\right)-\mathrm{E}\left(0\right)-\mathrm{M}\left(0\right)-\mathrm{C}\left(0\right)-{\mathrm{N}}_{\mathrm{H}}\left(0\right)-\mathrm{H}\left(0\right)-\mathrm{R}\left(0\right)-\mathrm{D}\left(0\right)=\mathrm{2,256,524}$; and in rural health district, $\mathrm{N}\left(0\right)=\mathrm{103,4370},\mathrm{ E}\left(0\right)=5310,\mathrm{ M}\left(0\right)=620,\mathrm{ C}\left(0\right)=200, {\mathrm{N}}_{\mathrm{H}}\left(0\right)=80,\mathrm{ H}\left(0\right)=25,\mathrm{ R}\left(0\right)=6,\mathrm{ D}\left(0\right)=0,\mathrm{ S}\left(0\right)=\mathrm{N}\left(0\right)-\mathrm{E}\left(0\right)-\mathrm{M}\left(0\right)-\mathrm{C}\left(0\right)-{\mathrm{N}}_{\mathrm{H}}(0)-\mathrm{H}\left(0\right)-\mathrm{R}\left(0\right)-\mathrm{D}\left(0\right)=\mathrm{1,028,129}$. Figure 4 shows the incidence data of COVID-19 (red dash) and the model fitted curve (blue solid curve).

**Table 1:** Depiction and estimation of the model parameters.

Parameters	Description	Metropolitan health district	Rural health district	References
N	Total population	2,265,170	1,034,370	^24^
$\upbeta$	Transmission rate	$1.00\times {10}^{-5}$	$2.87\times {10}^{-5}$	Fitted
$\mathrm{\alpha }$	Average period from E to M	1/5 ${\mathrm{day}}^{-1}$	1/5 ${\mathrm{day}}^{-1}$	Fitted
$\upphi$	Average period from M to C	1/7 ${\mathrm{day}}^{-1}$	1/7 ${\mathrm{day}}^{-1}$	^25^
$\upomega$	Disease related death rate	0.3	0.3	Assumed
$\uprho$	Average period from M to R	0.8451 ${\mathrm{day}}^{-1}$	0.845 $1 {\mathrm{day}}^{-1}$	Assumed
${\uptau }_{1}$	Average period from ${\mathrm{N}}_{\mathrm{H}}$ to R	1/42 ${\mathrm{day}}^{-1}$	1/42 ${\mathrm{day}}^{-1}$	^26^
${\uptau }_{2}$	Average period from H to R	1/21 ${\mathrm{day}}^{-1}$	1/21 ${\mathrm{day}}^{-1}$	^26^
${\upgamma }_{1}$	Average period from C to ${\mathrm{N}}_{\mathrm{H}}$	0.13 ${\mathrm{day}}^{-1}$	0.13 ${\mathrm{day}}^{-1}$	^25^
${\upgamma }_{2}$	Average period from C to H	0.555 ${\mathrm{day}}^{-1}$	0.555 ${\mathrm{day}}^{-1}$	Assumed
$\upmu$	Birth/death rate (inverse of life expectancy in Australia)	1/83 $yea{r}^{-1}$	1/83 $yea{r}^{-1}$	^27^
${\updelta }_{1}$	Average period from ${\mathrm{N}}_{\mathrm{H}}$ to D	$4.18\times {10}^{-4}$ ${\mathrm{day}}^{-1}$	$4.10\times {10}^{-4}$ ${\mathrm{day}}^{-1}$	Fitted
${\updelta }_{2}$	Average period from H to D	$3.83\times {10}^{-3}$ ${\mathrm{day}}^{-1}$	$3.63\times {10}^{-3}{\mathrm{day}}^{-1}$	Fitted

### Sensitivity of the model to parameters

**Figure 5:**
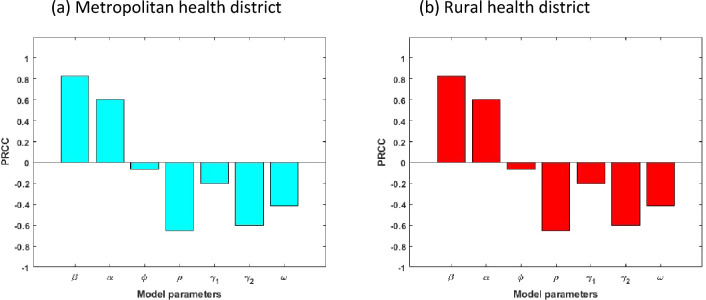
Correlation between Mild cases and the corresponding parameters of the model.

**Figure 6:**
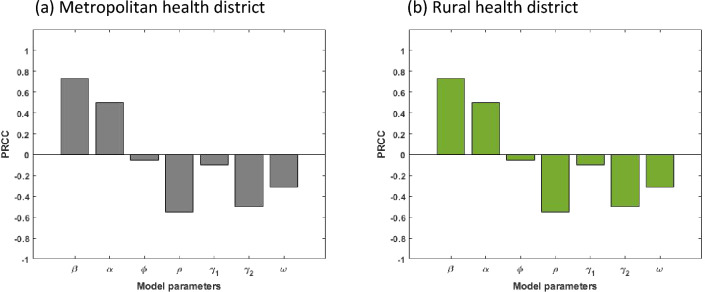
Correlation between Critical cases and the corresponding parameters of the model.

**Figure 7:**
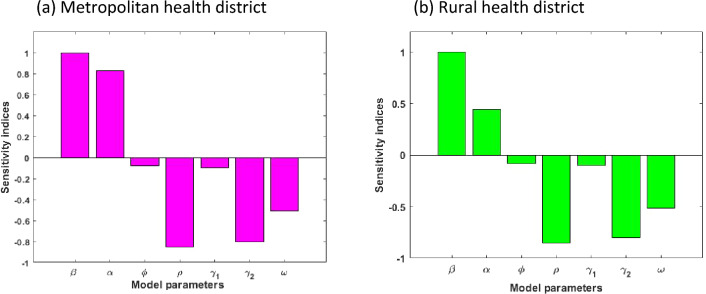
COVID-19 model sensitivities to its associated parameters of the model.

It is essential to discover how sensitive the COVID-19 model (2)–(9) is to variations in each of its parameters in order to advise intervention strategies that will support in bringing down the infection trajectory. Further, sensitivity analysis will help understand what should be prepared or avoided to mitigate the outbreak of the COVID-19^28,29^. For this purpose, we calculate the partial rank correlation coefficient (PRCCs)^30,31^ between each of the model parameters and several output variables (Mild and Critical cases) using a Latin Hypercube Sampling. Specially, a uniform distribution is allocated from half to fourfold baseline value (see Table 1) for each model parameters and assigned 100,000 simulations for each. Figures 5 and 6 display the correlations between Mild and Critical cases of metropolitan and rural health areas and the corresponding parameters $\upbeta ,\mathrm{ \alpha },\upphi ,\uprho , {\upgamma }_{1}, {\upgamma }_{2}$ and $\upomega$. From Figs. 5, 6 and 7, it is observed that Mild and Critical cases have a strong positive correlation with parameters $\upbeta$ (transmission rate) and $\mathrm{\alpha }$ (progression rate from E to M) in both metropolitan and rural health areas, implying that increasing $\upbeta$ and $\mathrm{\alpha }$ will rise Mild and Critical cases. On the other hand, parameters $\upphi ,\uprho , {\upgamma }_{1}, {\upgamma }_{2}$ and $\upomega$ have a negative correlation with Mild and Critical cases, implying that increasing those parameter values will reduce Mild and Critical cases.

As discussed in earlier sections, the scale and severity of COVID-19 transmission are directly related to the basic reproduction number ${\mathrm{R}}_{0}.$ Here, we assessed the sensitivity indices of the reproduction number ${\mathrm{R}}_{0}$. The indices identify how significant each parameter is to ${\mathrm{R}}_{0}$ and thus the COVID-19 transmission dynamics, and recognize which area should be focused in terms of intervention strategies.

Thus, from the explicit formula for ${\mathrm{R}}_{0}$, the analytical expression for the sensitivity indices can be derived as a comparative variation in ${\mathrm{R}}_{0}$ when each parameter changes using the following equation ^32^:

$${\Upsilon }_{\mathrm{q}}^{{\mathrm{R}}_{0}}= \frac{{\partial \mathrm{R}}_{0}}{\partial \mathrm{q}}\times \frac{\mathrm{q}}{{\mathrm{R}}_{0}},$$

where ${\Upsilon }_{\mathrm{q}}^{{\mathrm{R}}_{0}}$ is the sensitivity index of a differentiable ${\mathrm{R}}_{0}$ for any parameter, $\mathrm{q}$.

The sensitivity indices for the model (2)–(9) are graphically presented in Fig. 7. It can be perceived that of all the positives indices, the effective contact rate,$\upbeta$, is the highest in both metropolitan and rural areas, and therefore the most sensitive parameter. The value of the sensitive index suggests that an increase (or a decrease) in the value of $\upbeta$ will increases (or decrease) ${\mathrm{R}}_{0}$ by 100%. However, of all the negative indices displayed in Fig. 7, the recovery rate for the Mild class, denoted by $\uprho$, is the most sensitive parameter in both metropolitan and rural areas. An increase (or a decrease) of the value of $\uprho$ will decrease (or increase) ${\mathrm{R}}_{0}$ by 85%. Moreover, we observed that the progression rate $\mathrm{\alpha }$ is comparatively more sensitive in the metropolitan area than in the rural area.

**Figure 8:**
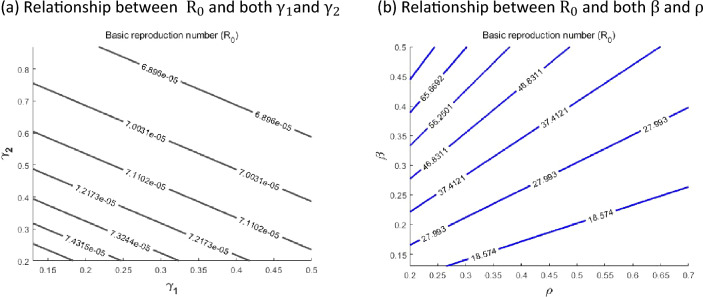
Contour plots of the basic reproduction number ${\mathrm{R}}_{0}$ with various values of other parameters.

Additionally, contour plots of ${\mathrm{R}}_{0}$ as a function of other parameters are displayed in Fig. 8 to determine how variations in these parameters affect the basic reproduction number, ${\mathrm{R}}_{0}$. Figure 8A shows a decrease in ${\mathrm{R}}_{0}$ with increasing recovery rates for both progression rates from Critical (C) class to Non-hospitalised and Hospital classes. Further, Fig. 8B shows a decreasing trend of the basic reproduction number with both transmission rate, $\beta$, and recovery rate from M to R, $\rho$. It is conjectured that ${R}_{0}$ can be brought lower than the threshold of one if efforts are geared towards dropping the contact rate while concurrently improving control and treatment of COVID-19 cases.

### Ethical approval

This study is based on aggregated COVID-19 surveillance data from the New South Wales (NSW) in Australia provided by the NSW government. No confidential information was included because mathematical analyses were performed at the aggregate level. We compiled data from the publicly available website https://data.nsw.gov.au/search/dataset/ds-nsw-ckan-aefcde60-3b0c-4bc0-9af1-6fe652944ec2/details?q = .

## Optimal control strategy and analysis

With consideration of the sensitivity result, control measures that were implemented to other diseases models^31–36^ were considered to see their effectiveness in controlling the spread of COVID-19 in the population of the metropolitan and rural area. This is executed by introducing two time-dependent control variables ${\mathrm{u}}_{1}(\mathrm{t})$ and ${\mathrm{u}}_{2}(\mathrm{t})$, defined as follows:

i. ${\mathrm{u}}_{1}(\mathrm{t})$ denotes the preventive strategy that is the effort at inhibiting the virus transmission and prevention of case development from Exposed, Mild, Critical, Non-hospitalised and Hospitalised population. This can be reached through public health advocacy for social distancing, good personal hygiene, diagnosis campaigns, wearing face masks in public places, education programs for public health, effective treatment with completion, and protective gear for healthcare workers. Noting that ${\mathrm{u}}_{1}\left(\mathrm{t}\right)=1$ indicates the policy effectively protects against infection, while ${\mathrm{u}}_{1}\left(\mathrm{t}\right)=0$ denotes the absence of the strategy.
ii. ${\mathrm{u}}_{2}(\mathrm{t})$ indicates control variable to enhance the management of Non-hospitalised and Hospitalised individuals with a view to ensure the rapid provision of additional treatment or oxygen or mechanical ventilation for Non-hospitalised and Hospitalised individuals with mild and severe COVID-19 symptoms. If ${\mathrm{u}}_{2}\left(\mathrm{t}\right)=1$, then the control strategy is effectively managing the disease, while ${\mathrm{u}}_{2}\left(\mathrm{t}\right)=0$ means the absence of the control strategy.

The forward–backward sweep method^37^ is used to solve the subsequent optimal control problems^38^. The incremental cost-effectiveness ratio (ICER) is used to determine the intervention strategy for the cost-effective analysis that is the best value for money. We provide detailed analysis of the optimal control of our proposed model in the supplementary materials (see the optimal control analysis section).

### Simulation of optimal control and cost-effective analysis

We implemented the Runge–Kutta fourth order forward and backward method using MATLAB programming language to solve the subsequent optimality system which consists of (Supplementary Eq. 12) and (Supplementary Eq. 16) with the characterization (Supplementary Eq. 18) within the period of [0, 100] days. The weight constants adopted for balancing the objective function (Supplementary Eq. 13) are selected to ensure that no term dominates the other. Therefore, we used equal weight constant for minimising the infectious classes, so that ${\mathrm{a}}_{1}={\mathrm{a}}_{2}={\mathrm{a}}_{3}={\mathrm{a}}_{4}={\mathrm{a}}_{5}=1.$ Under other conditions, the weight constants for determining efforts or cost essential to implement the controls are comparatively different, and outcomes in values for ${\mathrm{a}}_{6}=50$ and ${\mathrm{a}}_{7}=100$, which are consistent with previous research^39^. Details of the numerical procedure for simulating the obtained optimality system are contained^37^.

**Figure 9:**
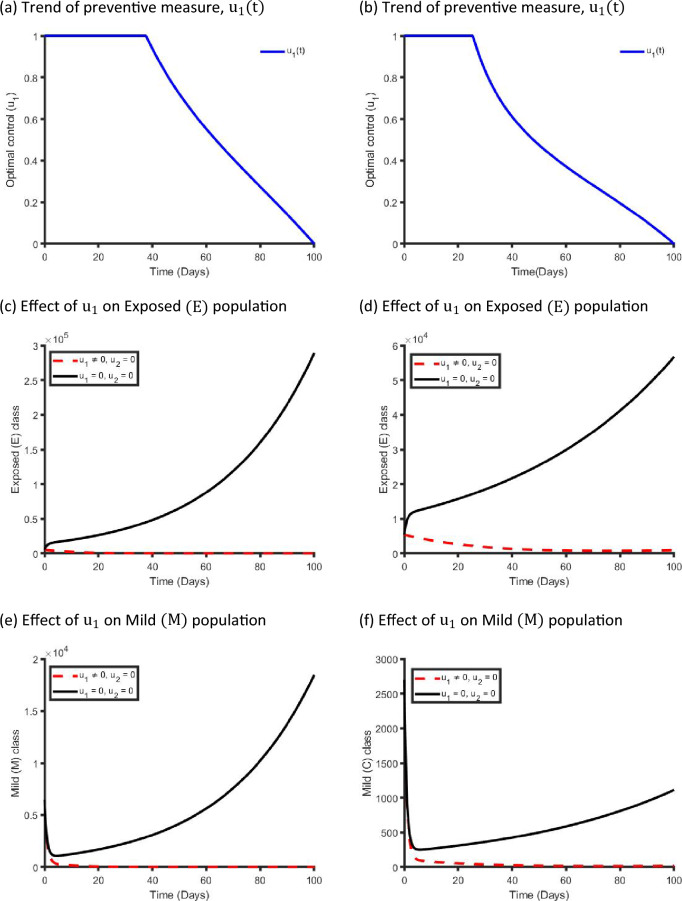

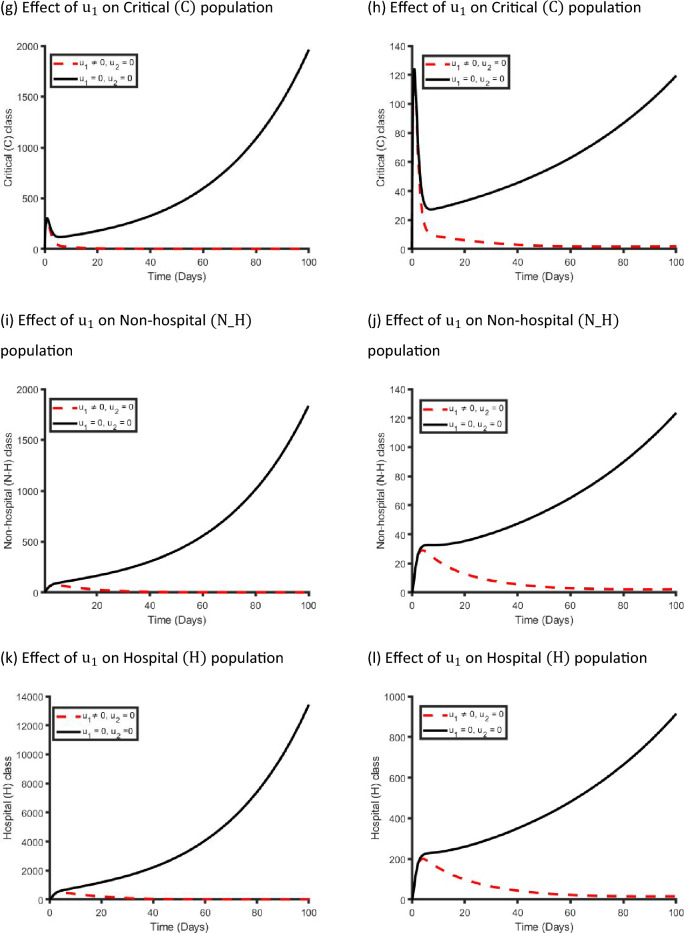
Control profile for preventive strategy (${\mathrm{u}}_{1}(\mathrm{t}))$ and its effects on the COVID-19 dynamics in metropolitan health districts (left hand side) and rural health districts (right hand side).

Figure 9 establishes how single preventive measure, ${\mathrm{u}}_{1}(\mathrm{t})$, affects the spread of the COVID-19 in the metropolitan and rural areas in NSW. As shown in Fig. 9a,b, to minimise the objective function (S13), the optimal control ${\mathrm{u}}_{1}(\mathrm{t})$ is continued at the maximum level (i.e. 100%) for about 40 days for metropolitan and 25 days for rural health districts before relaxing to the minimum in the final time. Also, as expected, the number of COVID-19 infectious individuals are reduced when control is in place.

**Figure 10:**
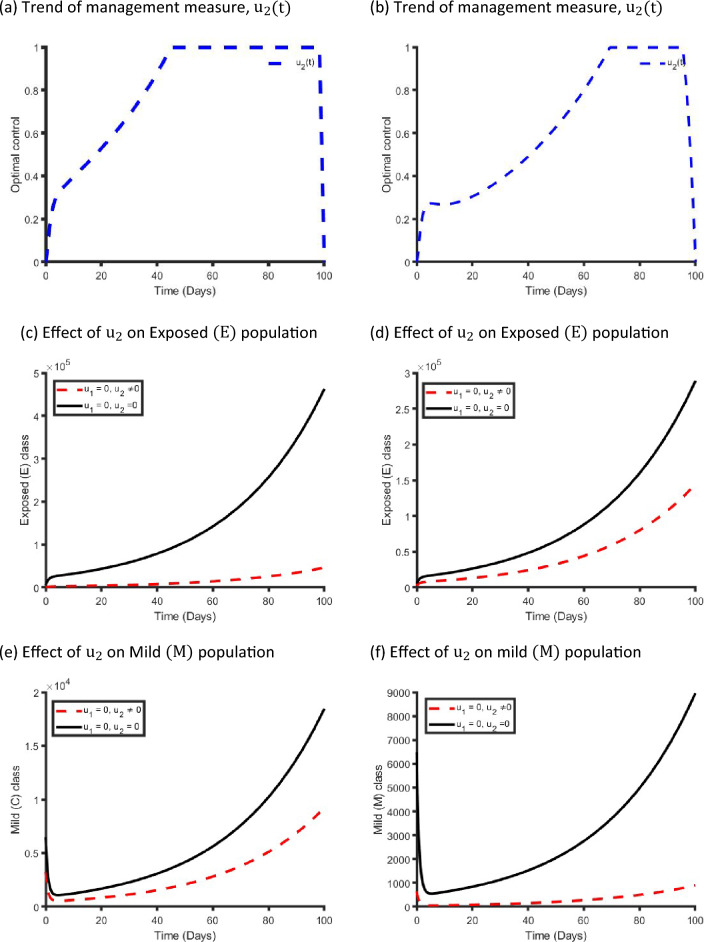

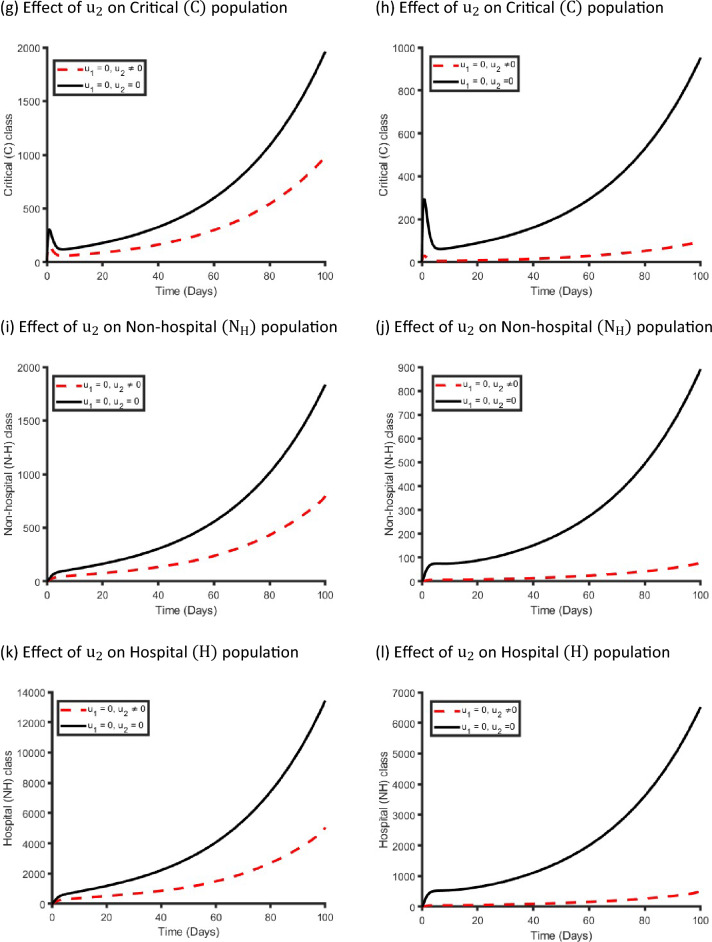
Control profile for management strategy (${\mathrm{u}}_{2}(\mathrm{t}))$ and its effects on the COVID-19 dynamics in metropolitan health districts (left hand side) and rural health districts (right hand side).

Figure 10 displays the effects of a single management intervention strategy ${\mathrm{u}}_{2}(\mathrm{t})$ on the dynamics of COVID-19 virus infection in the metropolitan and rural health districts. We observed that the ${\mathrm{u}}_{2}\left(\mathrm{t}\right)$ intervention has small impact on the Exposed, Mild and Critical class population while it has significant impact on Non-hospitalised and Hospitalised class population in both areas. Our finding is consistent with the reality because if we only take care of the Non-hospitalised and Hospitalised population, then the Critical class population moves to the Non-hospitalised and Hospitalised class population, and that is why implementing ${\mathrm{u}}_{2}(\mathrm{t})$ control strategy has significant impact on Non-hospitalised and Hospitalised class population.

**Figure 11:**
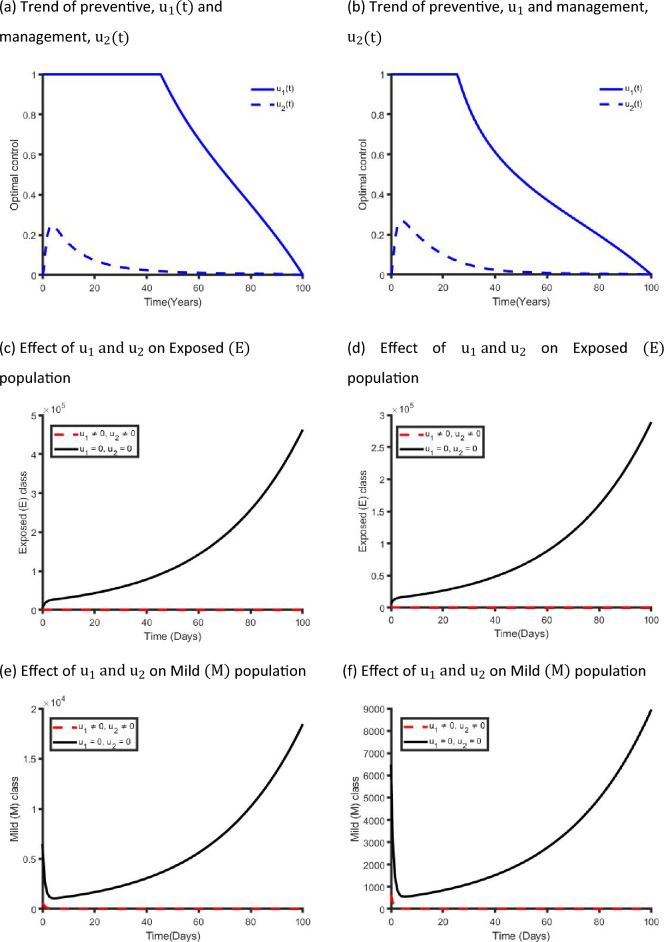

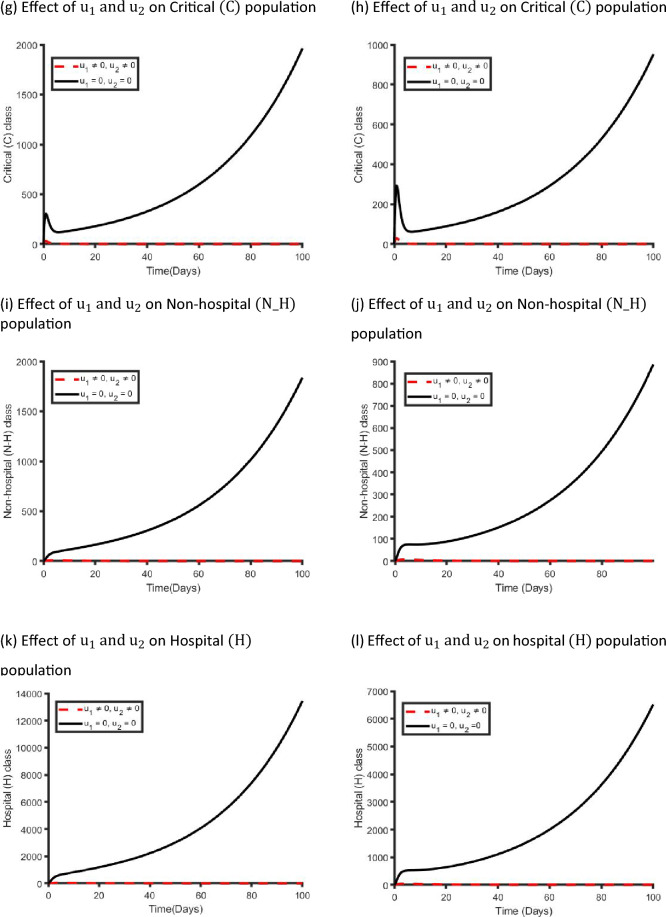
Control profile for preventive (${\mathrm{u}}_{1}(\mathrm{t}))$ and management (${\mathrm{u}}_{2}(\mathrm{t}))$ strategies and its effects on the COVID-19 dynamics in metropolitan health districts (left hand side) and rural health districts (right hand side).

Figure 11 shows the implication of combining the two optimal controls in bringing down the total number of infectious people to zero in both metropolitan and rural health areas. It is observed that optimal solution is achieved when preventive control strategy $({\mathrm{u}}_{1})$ is strictly adhered to at the maximum level of 100% for 45 days and 30 days, while the management control strategy $({\mathrm{u}}_{2})$ of the Non-hospitalised and Hospitalised individuals is at a maximum level of around 25% in metropolitan and rural health areas in NSW. It can be seen that the combination of the two control strategies is significantly more effective in decreasing the spread of the COVID-19 virus compared to the implementation of each control strategy individually. This is consistent with previous modelling studies^30,36,40^.

It is essential to determine the most cost-effective strategy among the single and combined control strategies to optimally mitigate the spread of COVID-19 at the possible minimum cost. This is performed by associating the differences among each intervention’s costs and outcomes; obtained by estimating the incremental cost-effective ratio (ICER), which is defined as the extra cost per additional intervention outcome. Incrementally, when analysing two or more competing intervention policies, one intervention is associated with the next less effective option. The ICER numerator is given by the total difference in intervention costs, active COVID-19 cases averted costs and averted productivity losses if applicable, between each scenario and baseline. The ICER denominator is the total number of active COVID-19 cases averted. Hence, the following formula obtains the ICER:

16 $$\mathrm{ICER}\,=\,\frac{\mathrm{Total\,cost}}{\mathrm{Total\,active\,cases\,averted}},$$

The total cost for each of the single implementation and mutual effort of the optimal control strategy is obtainable from the objective function (S13). The cases averted is invaded by computing the difference between infectious individuals with and without control strategy. Let, ${\mathrm{S}}_{1}, {\mathrm{S}}_{2}$ and ${\mathrm{S}}_{12}$ respectively represent single preventive intervention strategy ${\mathrm{u}}_{1}(\mathrm{t})$, single management control strategy ${\mathrm{u}}_{2}(\mathrm{t})$ and the combined effort of the two strategies. Table 2 summarises the ICER for each and the combination of the control variables ${\mathrm{u}}_{1}(\mathrm{t})$ and ${\mathrm{u}}_{2}(\mathrm{t})$ in increasing order of the total infection averted.

**Table 2:** ICER in the order of COVID-19 cases averted by control measures.

Areas	Control measures	Total infection averted	Total cost	ICER
Metropolitan	${\mathrm{S}}_{1}$	$2.182\times {10}^{6}$	$3.872\times {10}^{5}$	0.177
	${\mathrm{S}}_{2}$	$1.104\times {10}^{3}$	$8.899\times {10}^{5}$	815.127
	${\mathrm{S}}_{12}$	$2.186\times {10}^{6}$	$3.860\times {10}^{5}$	0.176
Rural	${\mathrm{S}}_{1}$	$7.003\times {10}^{5}$	$2.029\times {10}^{5}$	0.289
	${\mathrm{S}}_{2}$	$1.036\times {10}^{3}$	$7.848\times {10}^{5}$	757.528
	${S}_{12}$	$7.091\times {10}^{5}$	$2.020\times {10}^{5}$	0.284

The ICER results for ${\mathrm{S}}_{1}, {\mathrm{S}}_{2}$ and ${\mathrm{S}}_{12}$ are calculated using (16) and shown in Table 2 follows.

Comparing ${\mathrm{S}}_{1}, {\mathrm{S}}_{2}$ and ${\mathrm{S}}_{12}$, it is seen that combined intervention ${\mathrm{S}}_{12}$ is the most cost-effective which reduces a significant number of COVID-19 cases in both metropolitan and rural health areas compared to ${\mathrm{S}}_{1}$ and ${\mathrm{S}}_{2}$ individually, while ${\mathrm{S}}_{2}$ is the least effective intervention strategy among them.

## Discussion

COVID-19 is one of the most pressing public health problems in NSW. Overall, the transmission dynamics and epidemiology of COVID-19 in NSW is not entirely understood. The NSW government initiated various intervention programs to eliminate COVID-19 last year. Although COVID-19 control in NSW has remarkably progressed—accurate contact tracing, availability of free diagnostic and treatment services, the participation of many partners such as community health care agencies and general practitioners, newer diagnostic services, sufficient human resources, sufficient capacity e.g. hospital admission capacity, and guidelines—more effort is required. To reduce COVID-19 incidence and prevent deaths from COVID-19 in metropolitan and rural health districts of NSW, we need to identify the critical factors for developing COVID-19 disease, improve preventive and management strategies, treatment effectiveness, and reduce failure of treatment in infectious individuals.

In this study, we presented a mathematical analysis of transmission dynamics of the COVID-19 outbreak to deliver further understandings into the disease transmission and explore potential prevention and control strategies capable of reducing the disease spread at the metropolitan and rural districts for better health management in NSW. A compartmental mathematical model was formulated by subdividing the host population into Susceptible, Exposed, Mild, Critical, Non-hospitalised, Hospital, Recovered, and Death. The nonlinear mathematical model has been established by considering transmission routes from Exposed, Mild and Critical population individuals.

The formulated model was fitted to the reported data at the metropolitan and rural health districts in NSW, Australia. The least-square method was applied to estimates parameters such as transmission rate $(\upbeta )$ and progression rate $(\mathrm{\alpha })$ from Mild to Critical. The other parameters values were determined from the country demographic profile and literature review. We obtained the basic reproduction number $({\mathrm{R}}_{0})$, using the next generation matrix. Sensitivity analysis of the model was performed to find the parameters that drive the spread of the virus infection mostly in the population. It was revealed that transmission rate $(\upbeta )$ is the most sensitive parameter, which has a positive correlation with ${\mathrm{R}}_{0}$. It meant that decreasing the transmission will reduce the COVID-19 cases for both metropolitan and rural health geographies. Further, the recovery rate $(\uprho )$ is the second most sensitive parameter, negatively correlated with ${\mathrm{R}}_{0}$. It refers to increasing this parameter value will decrease the secondary cases of COVID-19.

We implemented an optimal control approach via Pontryagin’s Maximal principle^41^ and formulated the optimal strategies for controlling the COVID-19 epidemic at metropolitan and rural areas in NSW. Two different control strategies were considered including preventive strategy $({\mathrm{u}}_{1})$ and management of non-hospitalised and hospitalised strategy $({\mathrm{u}}_{2})$. Different settings were examined to measure the cost-effectiveness of both preventive and management control strategies. Between the two-single control strategies, the preventive strategy $\left({\mathrm{u}}_{1}\right)$ is better in cost-effectiveness than the management strategy $({\mathrm{u}}_{2})$ which reduce a significant number of COVID-19 cases in both metropolitan and rural health districts of NSW in Australia and similar to the previous study^26^. Therefore, our results suggest that the NSW government should improve preventive control interventions when only one control strategy is used. Naturally, this strategy actively decreases and/or stops the contact between susceptible and infectious individuals of COVID-19. However, combined implementation of preventive and management strategies is the most cost-effective measure for reducing the burden of COVID-19 in both metropolitan and rural districts in NSW.

Optimal control strategies have been applied in other endemic settings to minimise COVID-19 cases and intervention implementation costs. Previous studies show that preventive strategy is the best strategy for the single control strategy implementation to decrease COVID-19 cases and intervention costs^39,42,46^, which is similar to our results. However, our finding also suggests that combining control strategy with health management strategy, including enhanced services facilities and health management is the most effective way to decrease the COVID-19 burden in metropolitan and rural districts in NSW, consistent with previous works^39,41–45^.

Finally, in NSW, infectious disease surveillance is fully recognised, but the risk of bias cannot be precluded. More accurate data should be put in place to address concerns related to COVID-19. Accurate data leads to better estimation of crucial parameters, and this means our proposed intervention to decision support is data-dependent. Hence, local, state and federal level policy-makers need to adjust the possibility of under-reporting bias when investigating our outcomes. Therefore, more accurate data could be included in the model to explore the impact of preventive and management interventions on COVID-19 dynamics in NSW. Of the single intervention strategies, enhanced preventive strategy is cost-effective compared to management control strategy and prompt reducing COVID-19 cases in NSW. Yet, combining both preventive and management interventions is found to be even more cost-effective.

In summary, the paper provides mathematical modelling and optimal control strategy of COVID-19 in metropolitan and rural health districts of NSW for better pandemic management in Australia. We derived the basic reproduction number and found that it’s play an important role in the outbreak of COVID-19 in both districts. Sensitivity analysis also performed to identify the most important risk factors and found that transmission rate had largest influence on COVID-19 prevalence. We adopted optimal control analysis via Pontryagin’s Maximal Principle and formulated the optimal control strategies for controlling the COVID-19 outbreak in NSW, Australia. However, in our model we considered the total population size is constant and mixes homogeneously. That means that each individual in a compartment has identical disease susceptibility, infectiousness and transmission frequency with others, which neglects social behaviours of children, younger and older people that are known to be important risk factors in the spread of COVID-19. In addition, in our model individual-level COVID-19 natural history is not incorporated and not justified based on any empirical evidence. Therefore, future research could focus on individual-level data and quantifying any data-oriented reporting bias and modelling appropriate priority-based vaccination coverage.

Two different control strategies including preventive strategy and management control strategy were implemented to measure their cost-effectiveness for improved and consistent health service facilities to the communities. Among the two single-controls, the preventive control strategy is the most cost-effective. Therefore, when single control strategy is used, our results suggest that NSW government should improve preventive control strategy, reducing contact between infectious and susceptible individual. Our principal finding is that the combination of preventive and management control strategies such as the rapid provision of additional services means is the most impactful and cost-effectiveness strategy for reducing the spread of COVID-19 in NSW, Australia. The same modelling framework can be utilised for data obtained in other places around the globe.

## Supplementary Information

Supplementary Information.

## Data Availability

The datasets produced during the study are available from the corresponding author on reasonable request. All data were compiled from the publicly available website https://data.nsw.gov.au/search/dataset/ds-nsw-ckan-aefcde60-3b0c-4bc0-9af1-6fe652944ec2/details?q = .

## References

[1] Rahman A, Kuddus MA, Ip HL, Bewong M (2021). A review of COVID-19 modelling strategies in three countries to develop a research framework for regional areas. Viruses.

[2] NSW Health. Local health districts and specialty networks. New South Wales Health department. https://www.health.nsw.gov.au/lhd/Pages/default.aspx (accessed on 01/05/2022). (2018)

[3] Hawks L, Woolhandler S, McCormick D (2020). COVID-19 in prisons and jails in the United States. JAMA Intern. Med..

[4] Baraniuk C (2020). What the diamond princess taught the world about covid-19. BMJ.

[5] Finnie TJ, Hall IM, Leach S (2012). Behaviour and control of influenza in institutions and small societies. J. R. Soc. Med..

[6] Nowotny K, Bailey Z, Omori M, Brinkley-Rubinstein L (2020). COVID-19 exposes need for progressive criminal justice reform. Am. J. Public Health..

[7] Chao WC, Liu PY, Wu CL (2017). Control of an H1N1 outbreak in a correctional facility in central Taiwan. J. Microbiol. Immunol. Infect..

[8] Kayomo MK, Hasker E, Aloni M (2018). Outbreak of tuberculosis and multidrug-resistant tuberculosis, Mbuji-Mayi Central prison, democratic Republic of the Congo. Emerg. Infect. Dis..

[9] Ndeffo-Mbah ML, Vigliotti VS, Skrip LA, Dolan K, Galvani AP (2018). Dynamic models of infectious disease transmission in prisons and the general population. Epidemiol. Rev..

[10] Prem K, Liu Y, Russell TW (2020). The effect of control strategies to reduce social mixing on outcomes of the COVID-19 epidemic in Wuhan, China: A modelling study. Lancet Public Health..

[11] Shoukat A, Wells CR, Langley JM, Singer BH, Galvani AP, Moghadas SM (2020). Projecting demand for critical care beds during COVID-19 outbreaks in Canada. CMAJ.

[12] Rahman A, Kuddus MA (2021). Modelling the transmission dynamics of COVID-19 in six high-burden countries. Biomed. Res. Int..

[13] Nyabadza F, Chirove F, Chukwu CW, Visaya MV (2020). Modelling the potential impact of social distancing on the COVID-19 epidemic in South Africa. Comput. Math. Methods Med..

[14] Nyabadza F, Chirove F, Chukwu CW, Visaya MV (2020). Modelling the potential impact of social distancing on the COVID-19 epidemic in South Africa. Comput. Math. Methods Med..

[15] Truelove S, Abrahim O, Altare C (2020). The potential impact of COVID-19 in refugee camps in Bangladesh and beyond: A modeling study. PLoS Med..

[16] Abdulla F, Nain Z, Karimuzzaman M (2021). A non-linear biostatistical graphical modeling of preventive actions and healthcare factors in controlling COVID-19 pandemic. Int.’l J. Environ. Res. Public Health..

[17] Australia Government, Department of Health. Australia health sector emergency response plan for novel coronavirus (COVID-19).

[18] WHO. WHO timeline-COVID-19 (2020).

[19] Yang J, Zheng Y, Gou X (2020). Prevalence of comorbidities and its effects in patients infected with SARS-CoV-2: A systematic review and meta-analysis. Int. J. Infect. Dis..

[20] Diekmann O, Heesterbeek JA, Roberts MG (2010). The construction of next-generation matrices for compartmental epidemic models. J. R. Soc. Interface..

[21] van den Driessche P (2017). Reproduction numbers of infectious disease models. Infect. Dis. Model..

[22] Kuddus MA, McBryde ES, Adekunle AI, White LJ, Meehan MT (2022). Mathematical analysis of a two-strain tuberculosis model in Bangladesh. Sci. Rep..

[23] Government NSW. *Local health districts and specialty networks*. Retrieved from https://www.health.nsw.gov.au/lhd/Pages/default.aspx (2022).

[24] Locan government directory. Office of local government. Government of New South Wales. Estimated resident population (2020)

[25] World Health Organization. Report of the WHO-China joint mission on coronavirus disease 2019 (COVID-19) (Geneva) (2019).

[26] Yang P, Yang G, Qi J (2021). The effect of multiple interventions to balance healthcare demand for controlling COVID-19 outbreaks: A modelling study. Sci. Rep..

[27] Australian Institute of Health and Welfare. *Deaths in Australia.* Retrieved from https://www.aihw.gov.au/reports/life-expectancy-death/deaths-in-australia (2022).

[28] Kuddus MA, McBryde ES, Adekunle AI, White LJ, Meehan MT (2019). Mathematical analysis of a two-strain disease model with amplification. Chaos Solitons Fractals.

[29] Kuddus MA, Rahman A (2021). Analysis of COVID-19 using a modified SLIR model with nonlinear incidence. Results Phys..

[30] Alqarni MS, Alghamdi M, Muhammad T, Alshomrani AS, Khan MA (2020). Mathematical modeling for novel coronavirus (COVID-19) and control. Numer. Methods Partial Differ. Equ..

[31] Alzahrani EO, Ahmad W, Khan MA, Malebary SJ (2021). Optimal control strategies of Zika virus model with mutant. Commun. Nonlinear Sci. Numer. Simul..

[32] Olaniyi S (2018). Dynamics of Zika virus model with nonlinear incidence and optimal control strategies. Appl. Math. Inf. Sci..

[33] Asamoah JKK, Owusu MA, Jin Z, Oduro F, Abidemi A, Gyasi EO (2020). Global stability and cost-effectiveness analysis of COVID-19 considering the impact of the environment: Using data from Ghana. Chaos Solitons Fractals.

[34] Australian Institute of Health and Welfare. Deaths in Australia, AIHW, Australian Government, accessed 12 January 2023 (2022).

[35] Srivastav AK, Ghosh M, Li XZ, Cai L (2021). Modeling and optimal control analysis of COVID-19: Case studies from Italy and Spain. Math. Methods Appl. Sci..

[36] Kuddus MA, Meehan MT, White LJ, McBryde ES, Adekunle AI (2020). Modeling drug-resistant tuberculosis amplification rates and intervention strategies in Bangladesh. PLoS ONE.

[37] Choi W, Shim E (2021). Optimal strategies for social distancing and testing to control COVID-19. J. Theor. Biol..

[38] MATLAB V, 2017. 9.2. 0 (R2017a). *The MathWorks Inc: Natick, MA, USA*.

[39] Olaniyi S, Obabiyi O, Okosun K, Oladipo A, Adewale S (2020). Mathematical modelling and optimal cost-effective control of COVID-19 transmission dynamics. Eur. Phys. J. Plus.

[40] Chu Y-M, Farhan M, Khan MA (2021). Mathematical modeling and stability analysis of Buruli ulcer in Possum mammals. Results Phys..

[41] Pontryagin LS (1987). Mathematical Theory of Optimal Processes.

[42] Madubueze CE, Dachollom S, Onwubuya IO (2020). Controlling the spread of COVID-19: Optimal control analysis. Comput. Math. Methods Med..

[43] Lemecha Obsu L, Feyissa Balcha S (2020). Optimal control strategies for the transmission risk of COVID-19. J. Biol. Dyn..

[44] Alemneh HT, Telahun GT (2020). Mathematical modeling and optimal control analysis of covid-19 in Ethiopia. J. Imter. Math..

[45] Gatyeni SP, Chukwu CW, Chirove F, Fatmawati, Nyabadza F (2022). Application of optimal control to the dynamics of COVID-19 disease in South Africa. Sci Afr..

[46] Nyabadza F, Chirove F, Chukwu CW, Visaya MV (2020). Modelling the potential impact of social distancing on the COVID-19 epidemic in South Africa. Comput. Math. Methods Med..

