# Supplementary material for: Modelling COVID-19 pandemic control strategies in metropolitan and rural health districts in New South Wales, Australia
Source: Sci Rep. 2023 Jun 26;13:10352. doi: 10.1038/s41598-023-37240-8 (PMC10293262; doi:10.1038/s41598-023-37240-8)
Supplement: Supplementary file 1 — Supplementary Information. [file 41598_2023_37240_MOESM1_ESM.docx]

**Supplementary materials**

**Modelling COVID-19 pandemic control strategies in metropolitan and rural health districts in New South Wales, Australia**

Azizur Rahman^1*^, Md Abdul Kuddus^1, 2, 3^, Ryan H.L. Ip^1^ and Michael Bewong^1^

^1^School of Computing, Mathematics and Engineering, Charles Sturt University, Wagga Wagga NSW 2678, Australia

^2^Australian Institute of Tropical Health and Medicine, James Cook University, Townsville, QLD 4811, Australia

^3^Department of Mathematics, University of Rajshahi, Rajshahi-6205, Bangladesh

*Corresponding author: Dr. Azizur Rahman, School of Computing, Mathematics and Engineering, Charles Sturt University, Wagga Wagga, NSW 2678, Australia; Email: [azrahman@csu.edu.au](mailto:azrahman@csu.edu.au)

Existence of equilibria

Model equations

$\frac{\mathrm{dS}}{\mathrm{dt}}=\mu N-\beta\left( M+C \right)S-\mu S$ (1)

$\frac{\mathrm{dE}}{\mathrm{dt}}=\beta\left( M+C \right)S-(\alpha+\mu)E$ (2)

$\frac{\mathrm{dM}}{\mathrm{dt}}=\alpha E-(\phi+\mu+\rho)M$ (3)

$\frac{\mathrm{dC}}{\mathrm{dt}}=\phi M-(\omega+\mu+\gamma_{1}+\gamma_{2})C$ (4) $\frac{\mathrm{dN}_{H}}{\mathrm{dt}}=\gamma_{1}C-\left( \mu+\delta_{1}+\tau_{1} \right)N_{H}$ (5)

$\frac{\mathrm{dH}}{\mathrm{dt}}=\gamma_{2}C-(\mu+\delta_{2}+\tau_{2})H$ (6)

$\frac{\mathrm{dR}}{\mathrm{dt}}=\rho M+\tau_{1}N_{H}+\tau_{2}H-\mu R$ (7)

$\frac{\mathrm{dD}}{\mathrm{dt}}=\delta_{1}N_{H}+\delta_{2}H$ (8)

$N\left( t \right)=S\left( t \right)+E\left( t \right)+M\left( t \right)+C\left( t \right)+N_{H}(t)+H\left( t \right)+R\left( t \right)+D(t)$. (9)

Two types of equilibrium solutions in this system: the disease-free equilibrium, which is happen when the basic reproduction $R_{0}$ is less than one (i.e. $R_{0}<1$) and the endemic equilibrium, which is happened when $R_{0}>1$. We discuss these in order below

*Disease-free equilibrium* $(X_{0})$

It is clear from equations (1) – (8) that a disease-free equilibrium is always exists by applying

$E=M=C=N_{H}=H=R=D=0$. Hence the disease-free equilibrium point is given by

$X_{0}=\left( S_{0}, E_{0}, M_{0},C_{0}, {N_{H}}_{0} , H_{0}, R_{0}, D_{0} \right)$=$\left( N, 0, 0, 0, 0, 0, 0, 0 \right)$. (10)

*Endemic equilibrium* ($X^{*})$

Since system (1) – (7) are independent of equation (8), we can focus our attention on the reduce system (1) – (7). The endemic equilibrium point of the equations (1) – (7) is given by applying

$S\neq E\neq M\neq C\neq N_{H}\neq H\neq R\neq0$. Hence the endemic equilibrium point is given by

$X^{*}=\left( S^{*}, E^{*}, M^{*},C^{*}, N_{H}^{*}, H^{*}, R^{*} \right)$ where

$S^{*}=\frac{\left( \alpha+\mu\right)(\phi+\rho+\mu)(\omega+\gamma_{1}+\gamma_{2}+\mu)}{\alpha\beta(\omega+\gamma_{1}+\gamma_{2}+\mu+\phi)}$ ,

$E^{*}=\frac{\mu\left( R_{0}-1 \right)\left( \alpha+\mu\right)\left( \phi+\rho+\mu\right)^{2}\left( \omega+\gamma_{1}+\gamma_{2}+\mu\right)^{2}}{(\omega+\gamma_{1}+\gamma_{2}+\mu+\phi)\left( \left( \alpha+\mu\right)\left( \phi+\rho+\mu\right)\left( \omega+\gamma_{1}+\gamma_{2}+\mu\right) \right)}$ ,

$M^{*}=\frac{\alpha\mu\left( R_{0}-1 \right)\left( \alpha+\mu\right)\left( \phi+\rho+\mu\right)\left( \omega+\gamma_{1}+\gamma_{2}+\mu\right)^{2}}{(\omega+\gamma_{1}+\gamma_{2}+\mu+\phi)\left( \left( \alpha+\mu\right)\left( \phi+\rho+\mu\right)\left( \omega+\gamma_{1}+\gamma_{2}+\mu\right) \right)}$ ,

$C^{*}=\frac{\alpha\phi\mu(R_{0}-1)\left( \alpha+\mu\right)(\phi+\rho+\mu)(\omega+\gamma_{1}+\gamma_{2}+\mu)}{(\omega+\gamma_{1}+\gamma_{2}+\mu+\phi)\left( \left( \alpha+\mu\right)\left( \phi+\rho+\mu\right)\left( \omega+\gamma_{1}+\gamma_{2}+\mu\right) \right)}$ ,

$N_{H}^{*}=\frac{\alpha\phi\mu\gamma_{1} (R_{0}-1)\left( \alpha+\mu\right)(\phi+\rho+\mu)(\omega+\gamma_{1}+\gamma_{2}+\mu)}{(\delta_{1}+\tau_{1}+\mu)(\omega+\gamma_{1}+\gamma_{2}+\mu+\phi)\left( \left( \alpha+\mu\right)\left( \phi+\rho+\mu\right)\left( \omega+\gamma_{1}+\gamma_{2}+\mu\right) \right)}$ ,

$H^{*}=\frac{\alpha\phi\mu\gamma_{2} (R_{0}-1)\left( \alpha+\mu\right)(\phi+\rho+\mu)(\omega+\gamma_{1}+\gamma_{2}+\mu)}{(\delta_{2}+\tau_{2}+\mu)(\omega+\gamma_{1}+\gamma_{2}+\mu+\phi)\left( \left( \alpha+\mu\right)\left( \phi+\rho+\mu\right)\left( \omega+\gamma_{1}+\gamma_{2}+\mu\right) \right)}$ ,

$R^{*}=\left( \rho\left( \omega+\gamma_{1}+\gamma_{2}+u \right)+\frac{\phi\gamma_{1}\tau_{1}}{(\delta_{1}+\tau_{1}+\mu)}+\frac{\phi\gamma_{2}\tau_{2}}{(\delta_{2}+\tau_{2}+\mu)} \right)\frac{\alpha(R_{0}-1)\left( \alpha+\mu\right)(\phi+\rho+\mu)(\omega+\gamma_{1}+\gamma_{2}+\mu)}{(\omega+\gamma_{1}+\gamma_{2}+\mu+\phi)\left( \left( \alpha+\mu\right)\left( \phi+\rho+\mu\right)\left( \omega+\gamma_{1}+\gamma_{2}+\mu\right) \right)}$ (11)

Equation (11) shows that if $R_{0}>1$ then the endemic equilibrium $X^{*}\left( S^{*}, E^{*}, M^{*},C^{*}, N_{H}^{*}, H^{*}, R^{*} \right)\in D_{1}$.

Global stability of disease-free equilibrium (DFE)

Since our model equations from (1) – (7) are independent of the size of the Dead population D(t) ; therefore, if we only wish to track disease incidence and prevalence, we can consider the reduced system (1) – (7) for the global stability of disease-free equilibrium.

Theorem: The disease-free equilibrium of the model (1) – (7), given by (10), is globally asymptotically stable in $D_{1}$ whenever $R_{0}<1$.

Proof: We consider the following Lyapunov function,

$V_{0}=\left( S-S_{0}\mathrm{lnS} \right)+E+M+C+N_{H}+H+R+C_{1}$

where $C_{1}=-S_{0}+S_{0}\ln S_{0}$

Taking the derivative of $V_{0}(t)$ along system trajectories yields

$\dot{V}_{0}=\left( 1-\frac{S_{0}}{S} \right)\dot{S}+\dot{E}+\dot{M}+\dot{C}+\dot{N_{H}}+\dot{H}+\dot{R}$,

$$=\left( 1-\frac{S_{0}}{S} \right)\left( \mu N-\beta\left( M+C \right)S-\mu S \right)+\beta\left( M+C \right)S-\alpha E-\mu E+\alpha E-\phi M-\mu M-\rho M$$

$+\phi M-\omega C-\mu C-\gamma_{1}C-\gamma_{2}C+\gamma_{1}C-\mu N\_H-\delta_{1}N_{H}-\tau_{1}N_{H}+\gamma_{2}C-\mu H-\delta_{2}H-\tau_{2}H$

$+\rho M+\tau_{1}N_{H}+\tau_{2}H-\mu R$

Now, we substitute in the identity $\mu N=\mu S_{0}$ to obtain

$\dot{V}_{0}=\left( 1-\frac{S_{0}}{S} \right)\dot{S}+\dot{E}+\dot{M}+\dot{C}+\dot{N_{H}}+\dot{H}+\dot{R}$,

$$=\left( 1-\frac{S_{0}}{S} \right)\left( \mu S^{0}-\beta\left( M+C \right)S-\mu S \right)+\beta\left( M+C \right)S-\alpha E-\mu E+\alpha E-\phi M-\mu M-\rho M$$

$+\phi M-\omega C-\mu C-\gamma_{1}C-\gamma_{2}C+\gamma_{1}C-\mu N_{H}-\delta_{1}N_{H}-\tau_{1}N\_H+\gamma_{2}C-\mu H-\delta_{2}H-\tau_{2}H$

$+\rho M+\tau_{1}N_{H}+\tau_{2}H-\mu R$,

We can simplify this expression as,

$\dot{V}_{0}=\mu S_{0}\left( 2-\frac{S_{0}}{S}-\frac{S}{S_{0}} \right)+\beta S_{0}\left( M+C \right)-C\omega-\delta_{1}N_{H}-\delta_{2}H-\mu S_{0}$

We can simplify this expression further by substituting in the identity

$\beta\left( M+C \right)=\left( \alpha+\mu\right)\frac{E}{S}=\frac{(\alpha+\mu)(\phi+\mu+\rho)(\omega+\mu+\gamma_{1}+\gamma_{2})C}{\alpha\phi S}$ to get

$\dot{V}_{0}=\mu S_{0}\left( 2-\frac{S_{0}}{S}-\frac{S}{S_{0}} \right)+\frac{\left( \alpha+\mu\right)\left( \phi+\mu+\rho\right)\left( \omega+\mu+\gamma_{1}+\gamma_{2} \right)S_{0}C}{\alpha\phi S}-C\omega-\delta_{1}N_{H}-\delta_{2}H-\mu S_{0}$

$=\mu S_{0}\left( 2-\frac{S_{0}}{S}-\frac{S}{S_{0}} \right)+C\omega\left( \frac{\left( \alpha+\mu\right)\left( \phi+\mu+\rho\right)\left( \omega+\mu+\gamma_{1}+\gamma_{2} \right)}{\alpha\phi S\omega}-1 \right)-\delta_{1}N_{H}-\delta_{2}H-\mu S_{0}$

$=\mu S_{0}\left( 2-\frac{S_{0}}{S}-\frac{S}{S_{0}} \right)+C\omega\left( \frac{R_{0}}{S_{0}\alpha\beta\phi S\omega(\omega+\mu+\gamma_{1}+\gamma_{2}+\phi)}-1 \right)-\delta_{1}N_{H}-\delta_{2}H-\mu S_{0}$

$\dot{V}_{0} \leq\mu S_{0}\left( 2-\frac{S_{0}}{S}-\frac{S}{S_{0}} \right)+C\omega\left( R_{0}-1 \right)-\delta_{1}N_{H}-\delta_{2}H-\mu S_{0}$

Since the arithmetic mean is greater than or equal to the geometric mean. Therefore, the disease-free equilibrium is globally asymptotically stable if $R_{0}<1$.

Optimal control analysis

Subsequently, the optimal control model with the two time-dependent variables $u_{1}$ and $u_{2}$is given by the following non-linear differential equations:

$\frac{\mathrm{dS}}{\mathrm{dt}}=\mu N-(1-u_{1}(t))\beta\left( M+C \right)S-\mu S$

$\frac{\mathrm{dE}}{\mathrm{dt}}=(1-u_{1}(t))\beta\left( M+C \right)S-(\alpha+\mu)E$

$\frac{\mathrm{dM}}{\mathrm{dt}}=\alpha E-(\phi+\mu+\rho)M$

$\frac{\mathrm{dC}}{\mathrm{dt}}=\phi M-(\omega+\mu+\gamma_{1}+\gamma_{2})C$ (12)

$\frac{dN\_H}{\mathrm{dt}}=\gamma_{1}C-\left( \mu+\delta_{1} \right)N_{H}-\tau_{1}\left( 1+u_{2}\left( t \right) \right)N_{H}$

$\frac{\mathrm{dH}}{\mathrm{dt}}=\gamma_{2}C-(\mu+\delta_{2})H-{(1+u_{2}(t))\tau}_{2}H$

$\frac{\mathrm{dR}}{\mathrm{dt}}=\rho M+\tau_{1}\left( 1+u_{2}\left( t \right) \right)N_{H}+\tau_{2}(1+u_{2}(t))H-\mu R$

$\frac{\mathrm{dD}}{\mathrm{dt}}=\delta_{1}N_{H}+\delta_{2}H$.

The goal of presenting the two control variables is to seek the optimal solution required to minimise the numbers of Exposure, Mild, Critical, Non-hospitalised, Hospitalised individuals at minimum cost. Hence, the objective function for this optimal control problem is given by

$J \left( u_{1}^{*}, u_{2}^{*} \right)=\min_{0\leq u_{1}, u_{2}\leq1} \int_{0}^{T_{f}} \left( a_{1}E+a_{2}M+a_{3}C+a_{4}N_{H}+a_{5}H+\frac{1}{2}\left( a_{6}u_{1}^{2}\left( t \right)+a_{7}u_{2}^{2}(t) \right) \right)dt,$ (13)

where, constants $a_{i}, i=1, 2, \ldots, 7$ are positive weights essential to balance the objective function. Following other works on COVID-19 control problem ^1-4^, quadratic cost on the controls are chosen to ensure the control has only one extremum (i.e. maximum or minimum), where $\frac{1}{2}a_{6}u_{1}^{2}(t)$ is the total cost of executing the preventive measure, and $\frac{1}{2}a_{7}u_{2}^{2}(t)$ is the total cost of managing active cases over the time interval $\left[ T_{0}, T_{f} \right]$ (where initial time $T_{0}=0$, final time $T_{f}=100$ days period).

Precisely, the optimal control strategy $u^{*}=\left( u_{1}^{*}, u_{2}^{*} \right)$ is required such that

$J\left( u_{1}^{*}, u_{2}^{*} \right)=min\left\{ J\left( u_{1}, u_{2} \right):u_{1}, u_{2}\in U \right\}$, (14)

where, $U$ is the non-empty control set defined by

$U=\left\{ \left( u_{1}, u_{2} \right):\left( u_{1}\left( t \right), u_{2}\left( t \right) \right) are measurable with 0\leq u_{1}, u_{2}\leq1 for t\in[T_{0},T_{f}] \right\}$.

Thus, to regulate the necessary conditions that the optimal control strategy $\left( u_{1}^{*}, u_{2}^{*} \right)$ must satisfy the Pontryagin’s maximum principle ^5^, which changes into the control problem (14) subject to the model (12) that minimising pointwise a Hamiltonian $H_{1}$, with respect to the control measures. This Hamiltonian is given as

$H_{1}=a_{1}E+a_{2}M+a_{3}C+a_{4}N_{H}+a_{5}H+\frac{1}{2}\left( a_{6}u_{1}^{2}\left( t \right)+a_{7}u_{2}^{2}(t) \right)$

$+\lambda_{1}\left( \mu N-(1-u_{1}(t))\beta\left( M+C \right)S-\mu S \right)$

$+\lambda_{2}\left( (1-u_{1}(t))\beta\left( M+C \right)S-\alpha E-\mu E \right)$

$+\lambda_{3}\left( \alpha E-\phi M-\mu M-\rho M \right)$

$+\lambda_{4}\left( \phi M-\omega C-\mu C-\gamma_{1}C-\gamma_{2}C \right)$

$+\lambda_{5}\left( \gamma_{1}C-\mu N_{H}-\delta_{1}N_{H}-\tau_{1}\left( 1+u_{2}\left( t \right) \right)N_{H} \right)$

$+\lambda_{6}\left( \gamma_{2}C-\mu H-\delta_{2}H-{\left( 1+u_{2}\left( t \right) \right)\tau}_{2}H \right)$

$+\lambda_{7}\left( \rho M+\tau_{1}\left( 1+u_{2}\left( t \right) \right)N_{H}+\tau_{2}(1+u_{2}(t))H-\mu R \right)$

$+\lambda_{8}\left( \delta_{1}N_{H}+\delta_{2}H \right)$, (15)

where, $\lambda_{i}, i=1, 2, 3, \ldots, 8$, represent the adjoint variables associated with the state variables of the model (12). The expected outcome for minimising control problem as performed in ^4,6^ is adapted below. Now using Pontryagin’s maximum principle, we obtain the following theorem.

**Theorem:** Given that $\left( u_{1}^{*}, u_{2}^{*} \right)$ minimises the objective function (13) subject to the corresponding system (12), then the adjoint variables$\lambda_{i}, i=1, 2, 3, \ldots, 8$, satisfy the following system

$\frac{d\lambda_{1}}{\mathrm{dt}}=\lambda_{1}\left( \left( 1-u_{1} \right)\beta\left( M+C \right)+\mu\right)-\lambda_{2}\left( 1-u_{1} \right)\beta(M+C)$

$\frac{d\lambda_{2}}{\mathrm{dt}}= -1+\lambda_{2}\left( \alpha+\mu\right)-\lambda_{3}\alpha$

$\frac{d\lambda_{3}}{\mathrm{dt}}=-1+\lambda_{1}\left( \left( 1-u_{1} \right)\beta S \right)-\lambda_{2}\left( 1-u_{1} \right)\beta S+\lambda_{3}\left( \phi+\mu+\rho\right)-\lambda_{4}\phi-\lambda_{7}\rho$

$\frac{d\lambda_{4}}{\mathrm{dt}}=-1+\lambda_{1}\left( \left( 1-u_{1} \right)\beta S-\omega\right)-\lambda_{2}\left( 1-u_{1} \right)\beta S+\lambda_{4}\left( \omega+\mu+\gamma_{1}+\gamma_{2} \right)-\lambda_{5}\gamma_{1}-\lambda_{6}\gamma_{2}$ (16)

$\frac{d\lambda_{5}}{\mathrm{dt}}=-1+\lambda_{5}\left( \mu+\delta_{1}+\tau_{1}\left( 1+u_{2} \right) \right)-\lambda_{7}\tau_{1}\left( 1+u_{2} \right)-\lambda_{8}\delta_{1}$

$\frac{d\lambda_{6}}{\mathrm{dt}}= -1+\lambda_{6}\left( \mu+\delta_{2}+\tau_{2}\left( 1+u_{2} \right) \right)-\lambda_{7}\tau_{2}\left( 1+u_{2} \right)-\lambda_{8}\delta_{2}$

$\frac{d\lambda_{7}}{\mathrm{dt}}=\lambda_{7}\mu$

$\frac{d\lambda_{8}}{\mathrm{dt}}=\lambda_{8}\mu$

with the terminal (transversality) conditions

$\lambda_{i}\left( T_{f} \right)=0, i=1, 2, 3, \ldots, 8.$ (17)

Further, the optimal control pair $(u_{1}^{*}, u_{2}^{*})$ is given as follows

$u_{1}^{*}=max\left\{ 0, min\left\{ 1,\frac{\beta S(M+C)(\lambda_{2}-\lambda_{1})}{a_{6}} \right\} \right\}$,

$u_{2}^{*}=max\left\{ 0, min\left\{ 1,\frac{\left( \lambda_{5}-\lambda_{7} \right)\tau_{1}N\_H+\left( \lambda_{6}-\lambda_{7} \right)\tau_{2}H}{a_{7}} \right\} \right\}$ (18)

**Proof:** The existence of the optimal controls $u_{1}^{*}$ and $u_{2}^{*}$ such that

$J\left( u_{1}^{*}\left( t \right), u_{2}^{*} \right)={}_{U}^{\min}{J(u_{1}, u_{2})}$ with state system (12) is given by the convexity of the objective function integrand. By Pontryagin’s Maximum Principle ^5^, the adjoint equations and transversality conditions are obtained. Differentiation of Hamiltonian $H_{1}$ for the state variables gives the following system,

$\frac{d\lambda_{1}}{\mathrm{dt}}=-\frac{\partial H_{1}}{\partial S}$,

$\frac{d\lambda_{2}}{\mathrm{dt}}=-\frac{\partial H_{1}}{\partial E}$,

$\frac{d\lambda_{3}}{\mathrm{dt}}=-\frac{\partial H_{1}}{\partial M}$,

$\frac{d\lambda_{4}}{\mathrm{dt}}=-\frac{\partial H_{1}}{\partial C}$,

$\frac{d\lambda_{5}}{\mathrm{dt}}=-\frac{\partial H_{1}}{\partial N_{H}}$,

$\frac{d\lambda_{6}}{\mathrm{dt}}=-\frac{\partial H_{1}}{\partial H}$,

$\frac{d\lambda_{7}}{\mathrm{dt}}=-\frac{\partial H_{1}}{\partial R}$,

$\frac{d\lambda_{8}}{\mathrm{dt}}=-\frac{\partial H_{1}}{\partial D}$,

with $\lambda_{i}=0$, for $i=1, 2, 3, \ldots, 8$.

Optimal controls$u_{1}^{*}\left( t \right)$and $u_{2}^{*}\left( t \right)$are derived by the following optimality conditions,

$\frac{\partial H_{1}}{\partial u_{1}}=a_{6}u_{1}+\lambda_{1}\beta\left( M+C \right)S-\lambda_{2}\beta\left( M+C \right)S=0$,

$\frac{\partial H_{1}}{\partial u_{2}}=a_{2}u_{2}-\lambda_{5}\tau_{1}N_{H}-\lambda_{6}\tau_{2}H+\lambda_{7}\tau_{1}N_{H}+\lambda_{7}\tau_{2}H=0$,

at $u_{1}^{*}\left( t \right)$and $u_{2}^{*}\left( t \right)$ on the set $U$. On this set

$u_{1}^{*}\left( t \right)=\frac{\beta S(M+C)(\lambda_{2}-\lambda_{1})}{a_{6}}$ ,

$u_{2}^{*}\left( t \right)=\frac{\left( \lambda_{5}-\lambda_{7} \right)\tau_{1}N_{H}+\left( \lambda_{6}-\lambda_{7} \right)\tau_{2}H}{a_{7}}$.

This ends of the proof.

Supplementary References

1. Asamoah JKK, Owusu MA, Jin Z, Oduro F, Abidemi A, Gyasi EO, 2020. Global stability and cost-effectiveness analysis of COVID-19 considering the impact of the environment: using data from Ghana. *Chaos, Solitons & Fractals* 140: 110103.

2. Ullah S, Khan MA, 2020. Modeling the impact of non-pharmaceutical interventions on the dynamics of novel coronavirus with optimal control analysis with a case study. *Chaos, Solitons & Fractals* 139: 110075.

3. Srivastav A, Ghosh M, Li X, Cai L, 2020. Modeling and Optimal Control Analysis of COVID-19: Case Studies from Italy and Spain. *Authorea Preprints*.

4. Kuddus MA, Meehan MT, White LJ, McBryde ES, Adekunle AI, 2020. Modeling drug-resistant tuberculosis amplification rates and intervention strategies in Bangladesh. *Plos one* 15: e0236112.

5. Pontryagin LS, 2018. Mathematical theory of optimal processes. (Routledge).

6. Olaniyi S, Okosun K, Adesanya S, Lebelo R, 2020. Modelling malaria dynamics with partial immunity and protected travellers: optimal control and cost-effectiveness analysis. *Journal of biological dynamics* 14: 90-115.
